# Upper Limb Outcome Measures Used in Stroke Rehabilitation Studies: A Systematic Literature Review

**DOI:** 10.1371/journal.pone.0154792

**Published:** 2016-05-06

**Authors:** Leire Santisteban, Maxime Térémetz, Jean-Pierre Bleton, Jean-Claude Baron, Marc A. Maier, Påvel G. Lindberg

**Affiliations:** 1 Service de Médecine Physique et de Réadaptation, Université Paris Descartes, Hôpital Sainte-Anne, Paris, France; 2 FR3636 CNRS, Université Paris Descartes, Sorbonne Paris Cité, Paris, France; 3 Unité James Parkinson, service de Neurologie, Fondation OPH Rothschild, Paris, France; 4 Centre de Psychiatrie et Neurosciences, Inserm U894, Paris, France; 5 Université Paris Diderot, Sorbonne Paris Cité, Paris, France; University of Ottawa, CANADA

## Abstract

**Background:**

Establishing which upper limb outcome measures are most commonly used in stroke studies may help in improving consensus among scientists and clinicians.

**Objective:**

In this study we aimed to identify the most commonly used upper limb outcome measures in intervention studies after stroke and to describe domains covered according to ICF, how measures are combined, and how their use varies geographically and over time.

**Methods:**

Pubmed, CinHAL, and PeDRO databases were searched for upper limb intervention studies in stroke according to PRISMA guidelines and477 studies were included.

**Results:**

In studies 48different outcome measures were found. Only 15 of these outcome measures were used in more than 5% of the studies. The Fugl-Meyer Test (FMT)was the most commonly used measure (in 36% of studies). Commonly used measures covered ICF domains of body function and activity to varying extents. Most studies (72%) combined multiple outcome measures: the FMT was often combined with the Motor Activity Log (MAL), the Wolf Motor Function Test and the Action Research Arm Test, but infrequently combined with the Motor Assessment Scale or the Nine Hole Peg Test. Key components of manual dexterity such as selective finger movements were rarely measured. Frequency of use increased over a twelve-year period for the FMT and for assessments of kinematics, whereas other measures, such as the MAL and the Jebsen Taylor Hand Test showed decreased use over time. Use varied largely between countries showing low international consensus.

**Conclusions:**

The results showed a large diversity of outcome measures used across studies. However, a growing number of studies used the FMT, a neurological test with good psychometric properties. For thorough assessment the FMT needs to be combined with functional measures. These findings illustrate the need for strategies to build international consensus on appropriate outcome measures for upper limb function after stroke.

## Introduction

Stroke is a main cause of physical disability in adults [1]. Stroke survivors can suffer several neurologic impairments and up to 85% of them experience some degree of paresis of the upper limb [2]. Moreover, about 50% of stroke survivors show impaired upper limb and hand function in the chronic phase [3, 4]. This impairment often causes limitations in activities of daily living and may decrease the quality of life [5]. Although many studies have investigated the efficacy of various rehabilitation interventions, how best to improve upper limb function after stroke remains an important challenge.

Measurement of upper limb function is central for improving clinical practice and for evaluating efficacy of rehabilitation interventions. For the individual stroke patient, selection of an appropriate outcome measure can improve diagnosis and quantification of symptoms, aid planning and follow-up of rehabilitation interventions, and improve communication between clinicians [6]. Across patients, a standardized approach in the selection of outcome measures can lead to more efficient rehabilitation for the patient group and to greater insights into the clinical condition. However, many valid and reliable outcome measures for the upper limb exist and measures are often combined in order to gain a more complete picture of functioning [7–9]. Clinical practice guidelines for rehabilitation after stroke recommend use of outcome measures with good psychometric properties [10]. However, recommendations for which measures to use are not provided. Other guidelines recommended to select outcome measures that are appropriate to the interventions being studied and that are feasible in terms of time taken to administer and training of personnel [10]. Other practice guidelines suggest applying commonly used outcome measures with good psychometric properties, such as the Fugl-Meyer Test, the Action Research Arm Test, the Box and Block test, the Chedoke Arm and Hand Inventory, the Nine Hole Peg Test or the Wolf Motor Function Test [11]. A recent overview of systematic reviews on upper limb outcome measures showed good measurement quality for these same measures [7]. However, despite these guidelines studies performed still use a wide range of different outcome measures [12]. Most likely measures are selected depending on goals of study, severity of hemiplegia, whether patients are in acute or chronic phase, and preferences of the investigator [12, 13]. Nonetheless, investigators are generally aware of the weaknesses (and sometimes flaws) of each outcome measure, e.g., the presence of floor and ceiling effects [14] along with practical feasibility constraints (time taken and ease of administration) and develop study protocols around the limitations of existing outcome measures.

However, homogenous use of outcome measures is critical for across study comparison of the efficacy of different upper limb rehabilitation techniques. Representative meta-analyses require comparable, if not identical outcome measures. Yet, similar use of measures critically depends on whether scientists and clinicians agree on most appropriate measures. Some degree of consensus is expected, given that clinicians and scientists involved in designing studies are experts in the field. Guidelines and systematic reviews may contribute in forming consensus, but use of outcome measures in clinical studies still varies. A first step toward a more homogenous use of outcome measure requires anassessment of current use across performed studies. In this systematic review we aimed (i) to provide an overview of upper limb outcome measures used in interventional stroke studies and their frequency of use, (ii) to describe the different ICF domains covered by each measure, and (iii) to explore common patterns of use in terms of which measures are combined, and (iv) to examine whether their frequency of use is changing over time and whether use of measures varies between countries. The scope of this article does not include an overview of the psychometric properties of outcome measures since this has been reviewed recently [7, 15, 16]. We also limited this review to stroke and did not include other pathologies such as cerebral palsy where similar outcome measures are used [8].

## Methods

### Search strategy and selection criteria

**Fig 1:**
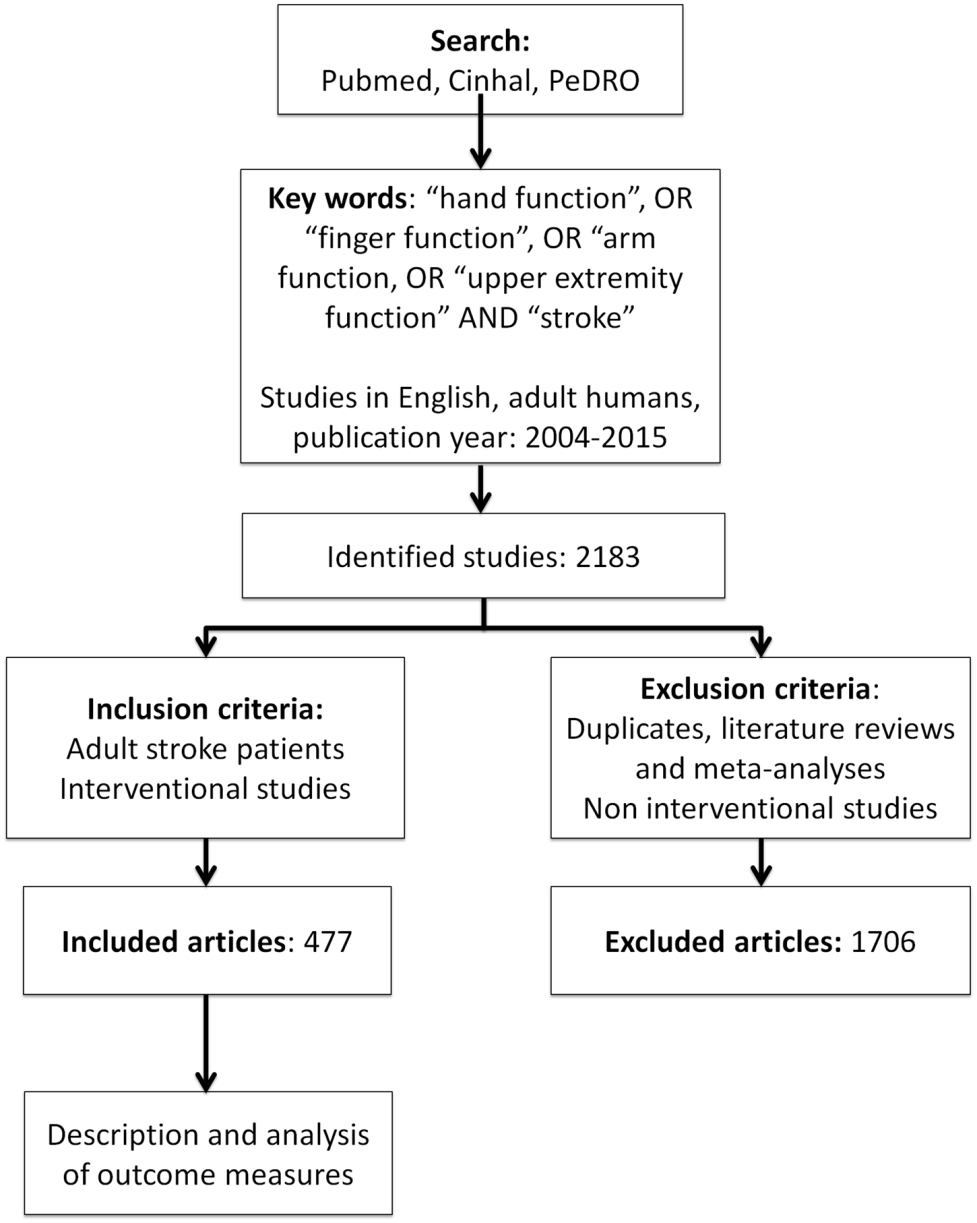
Flow-chart illustrating search strategy and number of studies selected.

A systematic literature search according to PRISMA guidelines [17] was performed from Pubmed, CinHal and PeDRO databases (S1 Text). The search keywords used were”Stroke”, AND “hand function”, OR”finger function”, OR “arm function”, OR “upper extremity function”. We limited the search to studies on human adults, written in English and published between December 2004 and August 2015. In total, 2183abstracts were identified and screened by four reviewers (LS, MT, JPB, PL). Only studies reporting on upper limb interventions were included. Non-interventional studies, such as cross-sectional studies or studies presenting new methods of assessment, were excluded. Duplicates, literature reviews and meta-analyses were also excluded. The included studies were not assessed for scientific quality. Full articles were reviewed when relevant information was lacking in the abstract (143 full articles checked). If inclusion was uncertain the study was discussed among reviewers and a final consensual decision was made whether to include or not. A total of477studies met criteria for inclusion and were reviewed for outcome measures (S1 Appendix). The selection steps are summarized in the flow-chart (Fig 1).

### Data analysis

All outcome measures used to assess the effects of upper limb interventions were recorded for each included study. Many studies applied instrumental measures such as electromyography, imaging, and transcranial magnetic stimulation and these measures were also recorded since these measures are becoming more common and more often integrated into clinical studies. Most studies combined upper limb outcome measures with other general measures (e.g., measures of stroke severity or disability). However, since these latter measures were not specific to upper limb function we did not include them in the analysis. For further analysis, various measures describing one given motor component were grouped. Force control (FC) measures included measures of power grip, pinch grip, release of force and force time integrals. Passive and active assessment of upper limb range of motion was also grouped into one variable (ROM). Similarly, the Ashworth scale and the modified Ashworth scales were grouped into one variable (Ashworth). In addition, the year of publication and the country where the study was performed were also recorded. The frequency of use of each outcome measure was then calculated as percent (%) across the studies included (n = 477).

Since many different outcome measures were identified, a second, more detailed analysis was undertaken for measures present in more than 5% of the studies. The items of each identified (or grouped) outcome measure were analyzed according to the International Classification of Functioning, Disability and Health framework (ICF) [18] by use of established linking rules [19]. Measures were categorized as mainly consisting of items relevant to (i) Body function and body structure, or (ii) Activity level. In addition, a further category was designated, (iii) Advanced methods, which consisted of methods mostly used in research and not yet commonly applied in clinical routine. If several upper limb outcome measures were used within a given study, we noted the frequency of combination between measures (i.e., how often measures were combined). Potential trends of use over time (2004–2014) were analyzed using the non-parametric Mann-Kendall Test. Finally, we also studied geographical trends by comparing frequency of use across different countries.

## Results

### Outcome measures: frequency of use

**Fig 2:**
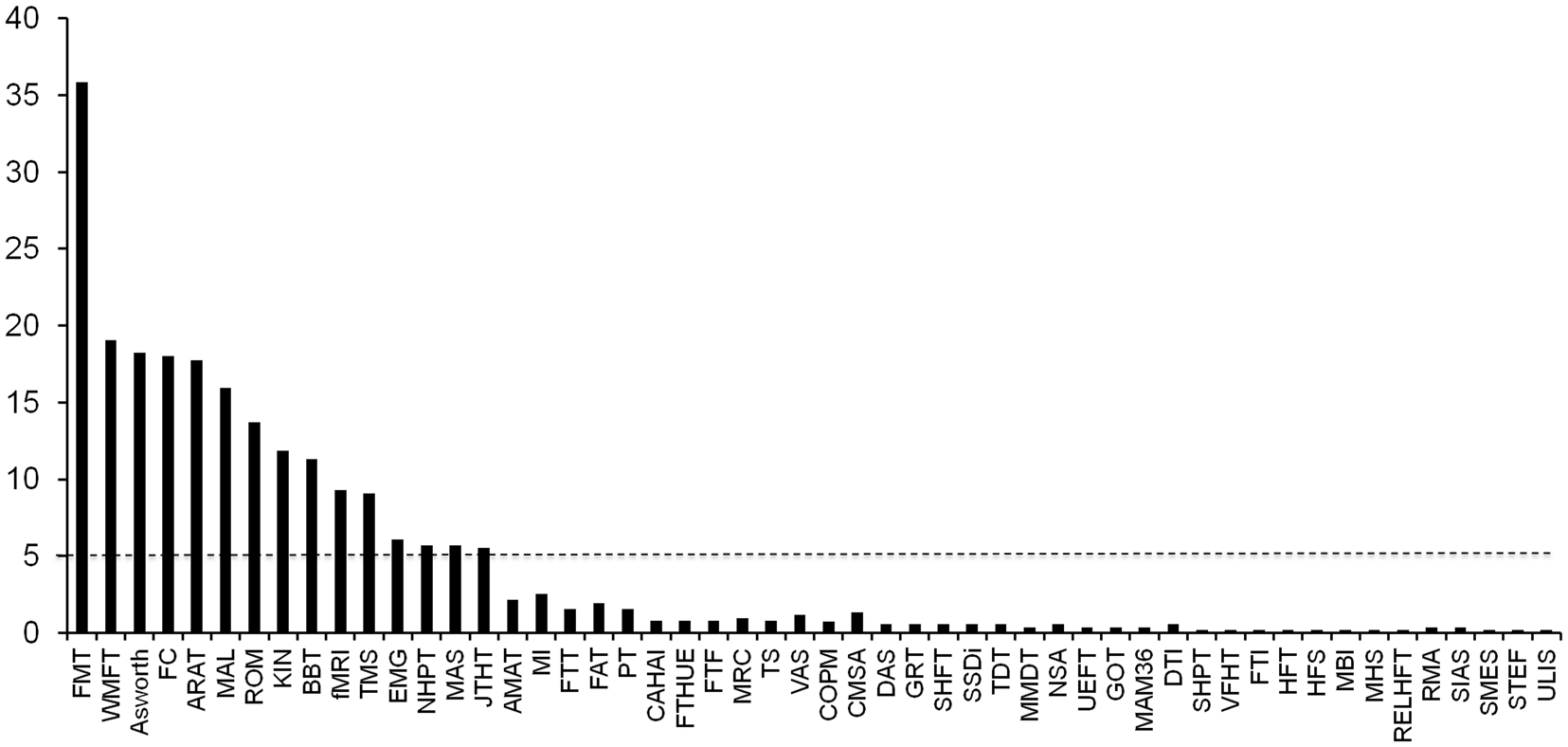
Frequency of use of different upper limb outcome measures (in % of studies). Frequency of use varies widely, between 36% and 1%. Only 15 measures were used in more than 5% of studies (dotted line). The 48 outcome measures are in alphabetic order: AMAT = Arm Motor Ability Test, ARAT = Action Research Arm Test, Ashworth = Ashworth scale, BBT = Box and Blocks Test, CAHAI = Chedoke Arm Hand Inventory, CMSA = Chedoke McMaster Stroke Assessment, COPM = Canadian Occupational Performance Measure, DAS = Disability Assessment Scale, DTI = Diffusion Tensor Imaging, EMG = Electromyography, FAT = Frenchay Arm Test, FC = Force Control, fMRI = Functional Magnetic Resonance Imaging, FMT = Fugl-Meyer Test, FTHUE = Functional Test for the Hemiplegic Upper Extremity, FTT = Finger Tapping Test, GOT = Grating Orientation Task, GRT = Grasp Release Test, HFS = Hand Function Survey, HFT = Hand Function Test, JTHT = Jebsen Taylor Hand Test, KIN = Kinematics, MAL = Motor Activity Log, MAM36 = Manual Ability Measurement 36, MAS = Motor Assessment Scale, MHS = Mini Hand Score, MI = Motricity Index, MMDT = Minnesota Manual Dexterity Test, NHPT = Nine Hole Peg Test, NSA = Nottingham Sensory Assessment, PT = Pegboard Test, RELHFT = Rehabilitation Engineering Laboratory Hand Function Test, RMA = Rivermead Motor Assessment, ROM = Range of Movement, SHFT = Shollerman Hand Function Test, SHPT = Sixteen Hole Peg Test, SIAS = Stroke Impairment Assessment Set, SMES = Sodring Motor Evaluation Scale, SSDI = Standardized Somatosensory Deficit Index, STEF = Simple Test for Hand Function, TDT = Tactile Discrimination Test, TMS = Transcranial Magnetic Stimulation, TS = Tardieu Scale, UEFT = Upper Extremity Function Test, ULIS = Upper Limb Impairment Scale, VAS = Visual Analogue Scale, VFHT = Von-Frey Hair Test, WMFT = Wolf Motor Function Test.

**Fig 3:**
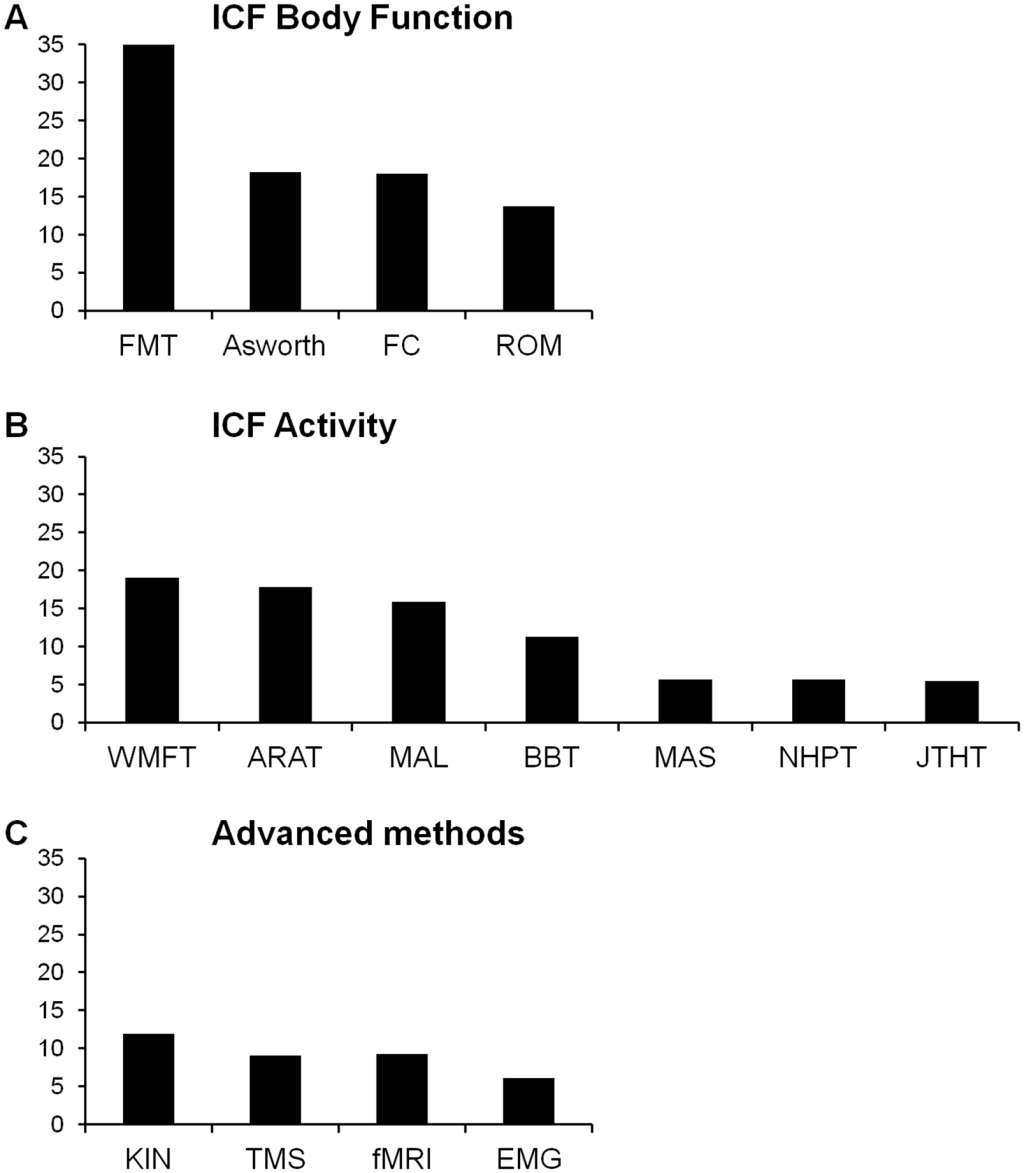
Frequency of use (%) of outcome measures according to ICF domains (A, B) and advanced methods (C). Abbreviations as in Fig 2.

A total of 477 studies were reviewed, in which a wide variety of outcome measures were identified. Interventions varied: 63% of studies used an active training paradigm (e.g., movement repetitions, strength training, use of robots), 24% of studies used a cognitive or sensory approach to enhance movement recovery (e.g., virtual reality games, mirror therapy, motor imagery), 25% combined approaches with conventional upper limb training, 11% of studies used pharmacological intervention (e.g., fluoxetine or botulinum toxin injection) and 10% of studies used neurostimulation (e.g., repetitive TMS or transcranial direct current stimulation). Fig 2 shows the distribution of the 48 identified measures in terms of frequency of use (% studies). Only the 15 most common measures were used in more than 5% of the studies. The most commonly used measure was the Fugl-Meyer test for the upper limb, reported in 36% of the studies. Fig 3 categorizes these15 top measures according to ICF or advanced methods. Four out of these 15 measures evaluated (entirely or predominantly) the ICF Body Structure/Body Function level (Fig 3A): the Fugl-Meyer Test (FMT), the Ashworth or modified Ashworth scale, tests concerning force control (FC) and ROM. Of these measures the Fugl-Meyer Test (FMT) was the most commonly used, almost twice as much as the others.

Seven of the 15 outcome measures concerned predominantly theICF Activity level. These were (Fig 3B): Wolf Motor Function Test (WMFT), Action Research Arm Test (ARAT), Motor Activity Log (MAL), and Box and Blocks Test (BBT), each of these present in about 10% of studies, followed by JebsenTaylor Hand Function Test (JTHFT), Nine Hole Peg Test (NHPT), and Motor Assessment Scale (MAS).

The remaining four of the 15 top measures were categorized as advanced methods. These include Transcranial Magnetic Stimulation (TMS), electromyography (EMG), functional MRI (fMRI) and Kinematics(KIN) (Fig 3C).

### Categorization of measure items according to ICF

Among the most commonly used measures (>5% of studies) some measures contained items that covered both ICF domains differently (Table 1). For example, the Fugl-Meyer Test (FMT) represents a mix, with most items (87%) related to the ICF Body Function/Body Structure domain (ICF B7, Musculoskeletal and Movement Related Functions), and some items (13%) related to the ICF Activity domain (D4, Mobility = 13%). In contrast, the Action Research Arm Test (ARAT)contains predominantly ICF Activity items (84%). Still other tests show a balanced distribution between the two ICF domains, such as the Wolf Motor Function Test (WMFT) and the Motor Assessment Scale (MAS). The Motor Activity Log (MAL) was the only one to include items on domestic activities (D6).

**Table 1:** Outcome measures in relation to ICF domains.

(%)	FMT	Ash-worth	FC	ROM	WMFT	MAL	ARAT	BBT	JTHT	MAS	NHPT
**D4. Mobility**	13.3	0	0	0	50	26.9	84.2	100	100	44.4	100
**D5. Self care**	0	0	0	0	0	50	0	0	0	0	0
**D6. Domestic life**	0	0	0	0	0	32.1	0	0	0	0	0
**B7.Movement functions**	86.6	100	100	100	50	0	15.8	0	0	55.6	0

ICF activity domains included: D4. Mobility, D5. Self care, D6. Domestic life.ICF body function/body structure domains included: B7. Musculoskeletal and movement related functions. ARAT = Action Research Arm Test, Ashworth = Ashworth scale, BBT = Box and Blocks Test, FC = Force Control, FMT = Fugl-Meyer Test, JTHT = Jebsen Taylor Hand Test, MAL = Motor Activity Log, MAS = Motor Assessment Scale, NHPT = Nine Hole Peg Test, ROM = Range of Movement, WMFT = Wolf Motor Function Test.

### Use of several outcome measures

**Fig 4:**
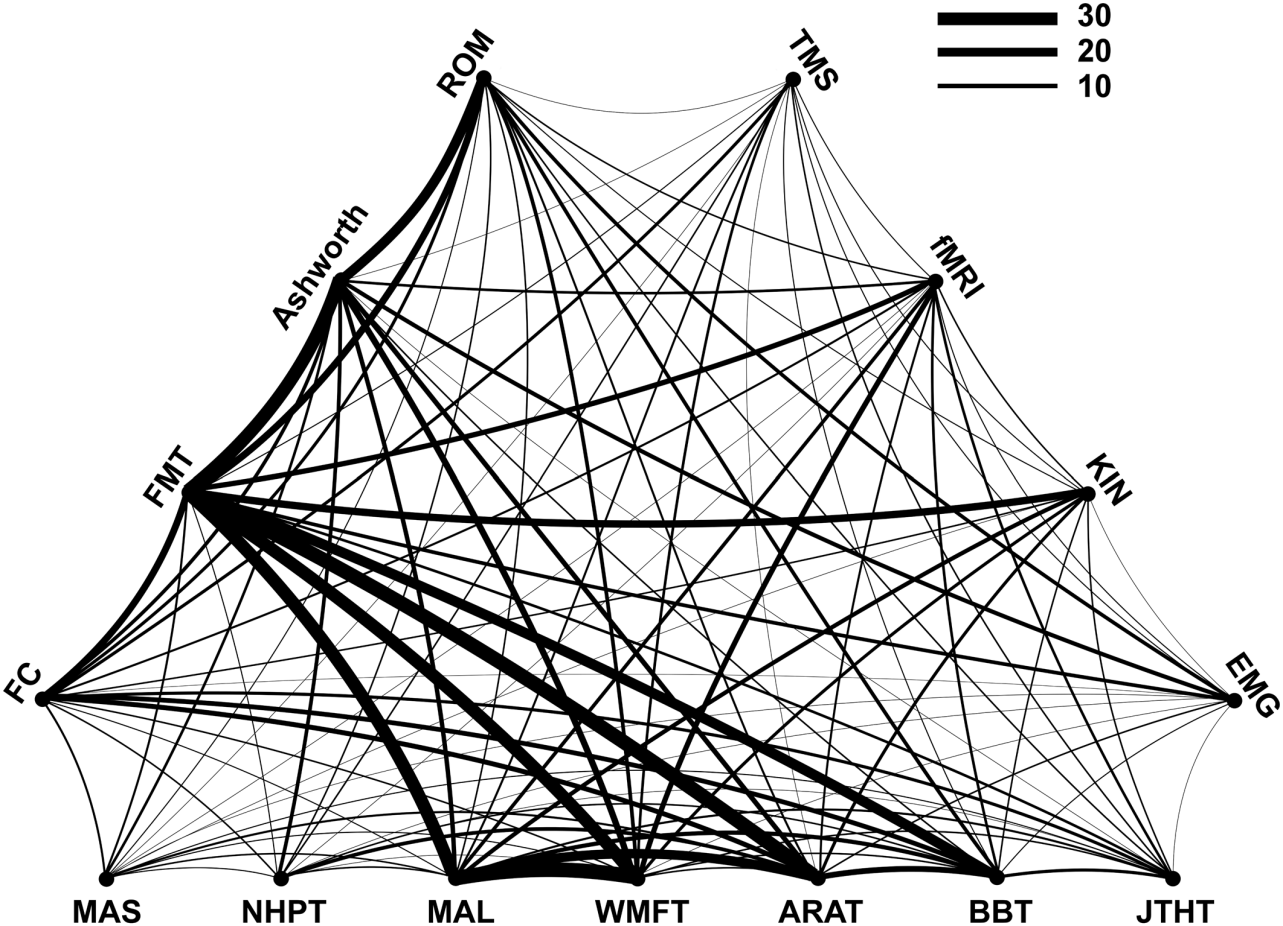
Combination of outcome measures. Measures related to the ICF Activity level are listed on the horizontal, those related to ICF Body function are shown on the left-top side, and those qualified as ‘Advanced methods’ are shown on the right-top side of the triangle. A curved link (line) between two different measures indicates their combined use within a study. The thickness of the curved line represents the frequency of occurrence across studies of a given combination.

In the majority (72%) of the studies, more than one upper limb outcome measure was used. Thirty-one percent of all studies combined two complementary measures and 25% combined three measures. Few studies used more than three measures, i.e. four or five were used by 11% and 4% of the studies, respectively. The particular combination of measures within studies (curved link) and the frequency of occurrence of particular combinations across studies (line thickness of the link) are shown in Fig 4. Combined use of the Motor Activity Log (MAL)and the Wolf Motor Function Test (WMFT)occurred most frequently. Similarly frequent was the association of the Fugl-Meyer Test (FMT) with the Action Research Arm Test (ARAT). Fig 4 also shows that many of the more frequent associations concern combinations between the two ICF domains. The Fugl-Meyer Test (FMT), related to the ICF Body Function/Body Structure level, was commonly combined with the measures related to ICF Activity level, such as the Motor Activity Log (MAL), the Wolf Motor Function Test (WMFT) and the Action Research Arm Test (ARAT). In contrast, certain measures were infrequently combined, e.g., the Fugl-Meyer Test (FMT) with either the Motor Assessment Scale (MAS) or the Nine Hole Peg Test (NHPT). Among the ‘advanced methods’, Kinematics (KIN) was most often combined with the Fugl-Meyer Test (FMT).

### Change over time

**Fig 5:**
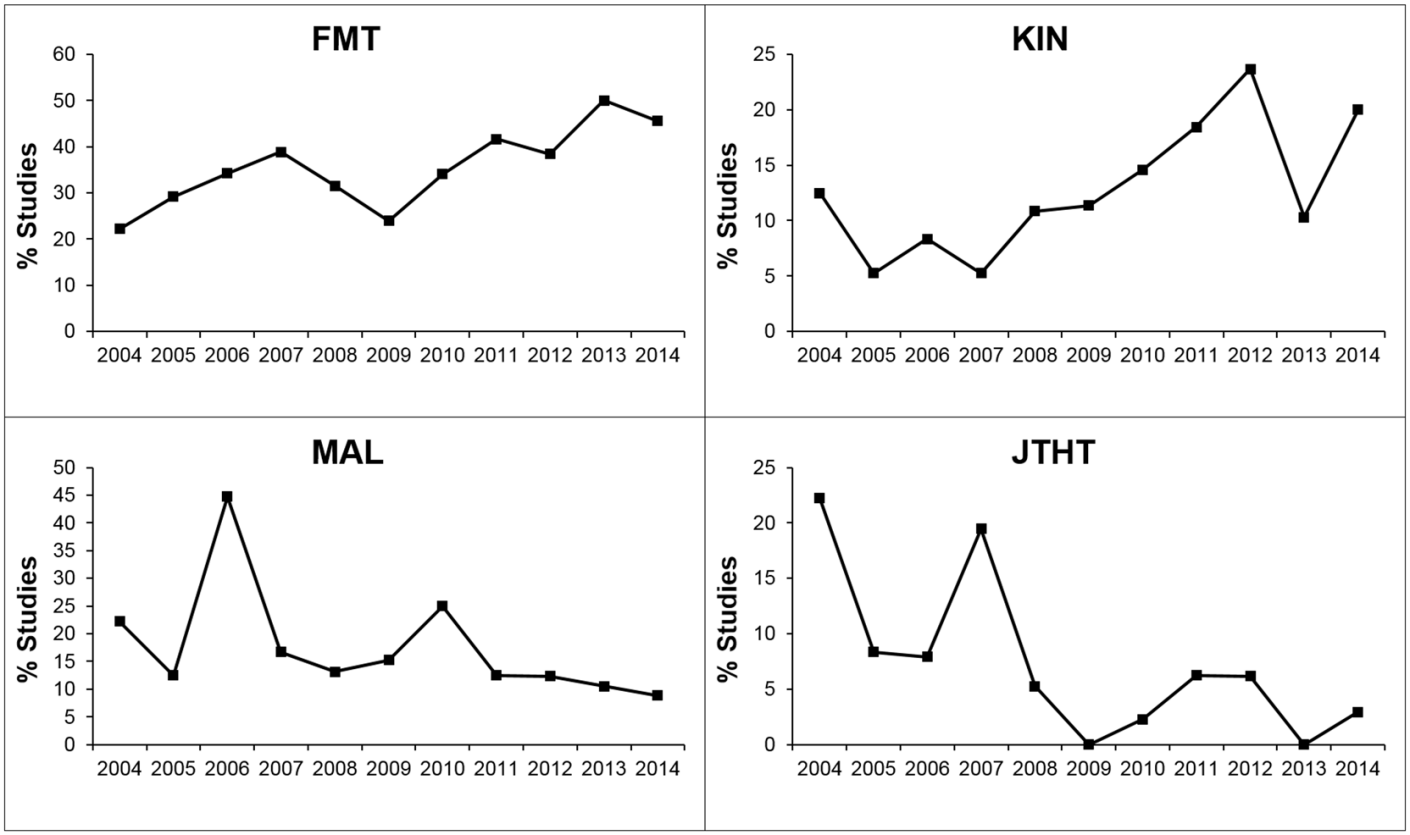
Increasing and decreasing frequency of use of outcome measures. The FMT and KIN both showed significantly increasing trends of use according to Mann-Kendall test (P<0.05, two-tailed). The MAL and the JTHT showed decreasing trends of use (P<0.05, two-tailed).

We used the Mann-Kendall test to statistically assess if there was a monotonic upward or downward trend in the frequency of use of the outcome measures over time. The Fugl-Meyer Test (FMT) showed an increasing trend of use across this twelve year period, with use in 30% of studies in 2004–2009 and 41% in 2010–2015 (Fig 5). There was also a trend for increased use of kinematics during this period (from 8% to 15%). The opposite tendency, a decrease in use, was found for the Motor Activity Log and the Jebsen Taylor Hand Test (MAL, from 21% to 13%; JTHT, from 8% to 4%, Fig 5).

### Geographical patterns of use

**Fig 6:**
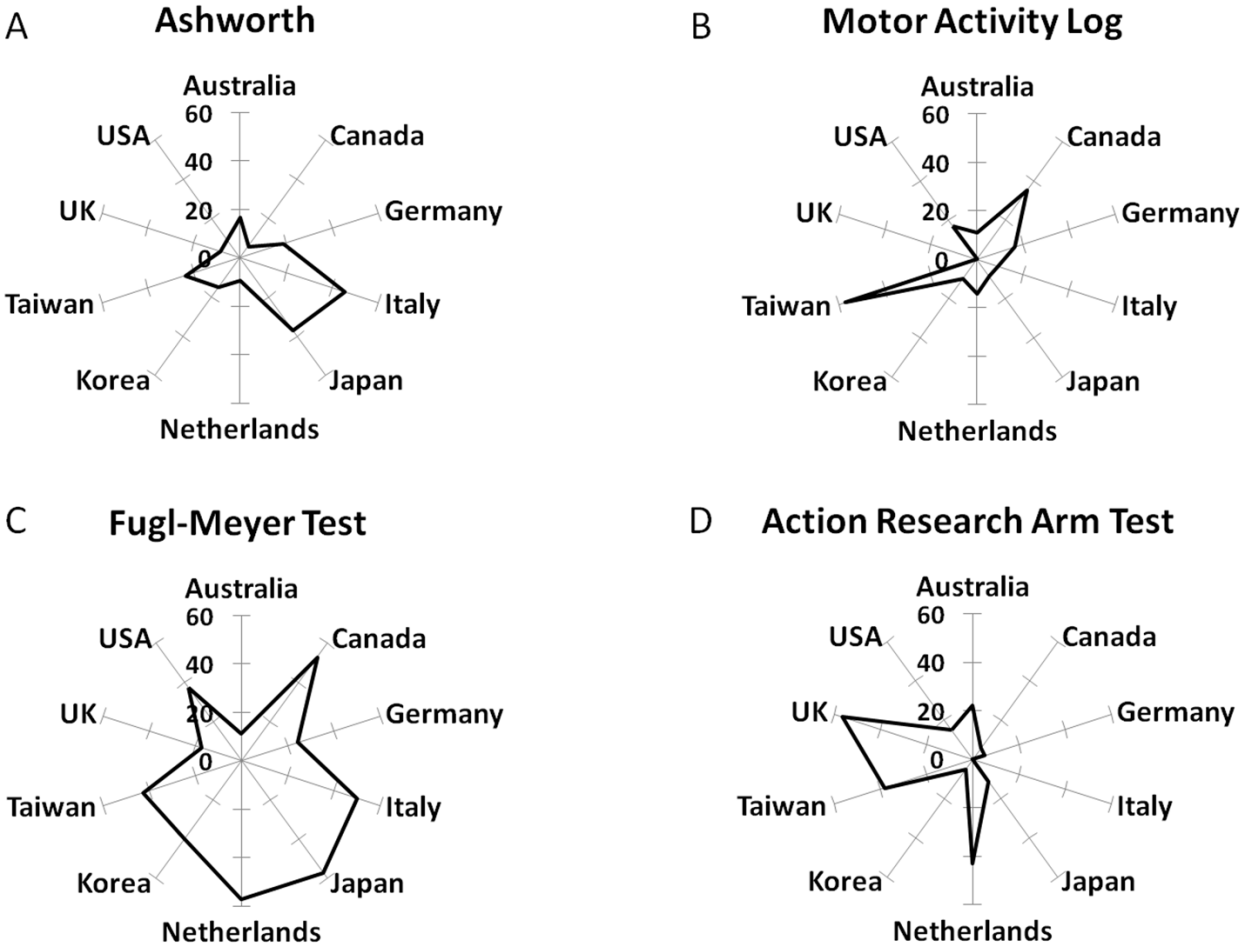
Frequency of use in the ten countries with most publications. Distributions (in % use) shown for Ashworth, Motor Activity Log (MAL), Fugl-Meyer Test (FMT) and Action Research Arm Test (ARAT). Frequency of use of particular measures across countries varied greatly and was not homogenous. The Fugl-Meyer Test (FMT, Fig 5C) has high rates of use in most countries, except in Australia and UK, where the Action Research Arm Test (ARAT, Fig 5D) was used more often.

The use of particular upper limb outcome measures may vary geographically. This was investigated for the 10 countries that published most of the included studies. The number of studies in these countries was: USA n = 169, Germany n = 37, Japan n = 35, UK n = 23, Italy n = 22, Taiwan n = 21, Netherlands n = 21, Republic of Korea n = 20, Australia n = 18, and Canada n = 17. The Fugl-Meyer Test (FMT) was the most used measure in studies from Canada (53%), Italy (50%), Japan (57%), Netherlands (57%) and USA (37%). Furthermore, the Fugl-Meyer Test (FMT) had the most homogenous pattern of use across the 10 countries and was among the top three measures in eight of these countries (Fig 6). Nonetheless, the Fugl-Meyer Test (FMT) was infrequently used in studies from the UK (17%) and Australia (11%). Other measures, such as Ashworth, Motor Activity Log (MAL) and Action Research Arm Test (ARAT), showed greater variation across countries with a less homogenous pattern of use (Fig 5). Studies from the UK used mostly the Action Research Arm Test (ARAT) (56%), Force Control (FC) (22%) and Range of Movement (ROM) (17%) measures, whereas studies from Australia used predominantly (44%) the Motor Assessment Scale (MAS), Range of motion measures (ROM, 28%) and the Action Research Arm Test (ARAT, 22%). Use of ‘Advanced methods’ appeared in the top three frequencies in the Republic of Korea (fMRI in 25% of studies), Japan (TMS in 20% of studies), and Germany (TMS in 24% of studies).

## Discussion

This review showed a large diversity of upper limb outcome measures used in intervention studies after stroke. This likely reflects differing priorities among scientists and clinicians when selecting outcome measures with choices influenced by several factors, such as level of residual voluntary motor capacity in the patient population, measurement properties of outcome measures (e.g., sensitivity and objectivity), feasibility for the study (time, personnel), and personal preferences. However, this study did reveal certain trends and patterns of use. The most frequently used measure was the Fugl-Meyer Test (FMT) and the findings showed how different measures were combined, how their use changed over time, and how their use differed between countries.

### The Fugl-Meyer Test: the most used outcome measure

The FMT was the most frequently used outcome measure, applied in 36% of the studies. This confirms previous reports that the FMT is the most commonly used measure when assessing upper limb function in stroke[9]. The FMT was developed in the 1970’s for measurement of sensorimotor impairments after stroke [20] based on the assumption that recovery after stroke follows a determined sequence. There is an upper and lower limb section covering reflexes, sensory and motor functions, balance, coordination and range of motion [21]. The upper limb section is often used separately in stroke studies describing upper limb function. The FMT mostly measures Body Functions and Body Structures according to ICF (Table 1). In the surveyed studies, the FMT was most often combined with the MAL, the WMFT and the ARAT. The MAL is a self-reporting assessment on how patients feel about their use of the affected upper limb in activities of daily living, thus this measure informs on the transfer of rehabilitation outcomes into everyday life. The ARAT measures hand and finger function in grasping and the WMFT, which includes timed, functional ability and strength parts, provides a description of upper limb function in the complementary ICF Activity domain. Given the varying degrees of severity and the heterogeneity of symptoms across patients, the combination of outcome measures is required for optimal description of upper limb status.

Advantages of the FMT include its feasibility (clinical application), validity and reliability [7, 22]. The minimally clinically important difference ranges from about 4–7 points, depending on sub-scale [23]. The psychometric properties of the FMT are sufficiently convincing that expert panels recommend use of this measure in clinical studies [15]. The good measurement properties and widespread knowledge of this measure likely explain why the FMT is the most commonly used measure. We found an increasing trend of use over time, with more recent studies using the FMT compared to studies performed 6–12 years ago. This suggests a growing consensus that this measure is appropriate for studies in stroke. Interestingly, the ARAT was also used frequently in certain countries (UK, Australia, Netherlands). The ARAT developed in the 1980s [24] has good psychometric properties [25]. A possible reason of the choice of this measure in clinical studies is that it is a quick and easy measure to use that informs about grasping abilities and some fine finger manipulation tasks.

Outcome measure selection varied across countries. Over 40% of studies from Canada, Italy, Japan, Netherlands, Republic of Korea and Taiwan included the FMT whereas only 17% of studies from the UK or 11% of studies from Australia included the FMT. Studies performed in these latter countries included other scales more often (such as ARAT and MAS). Interestingly, it was not the case that countries close by geographically or with the same language chose the same measures, e.g., differences in Ashworth use between Japan and Republic of Korea or differences in FMT use between USA and UK (Fig 6). Whether these different preferences constitute a geographical variation in opinions on how best to measure upper limb function warrants further study.

### ICF and upper limb function: match or mismatch?

A high degree of manual dexterity represents a hallmark of human (and primate) upper limb function. Upper arm control, such as in reach, i.e., transporting the hand to the object, is a prerequisite for dexterity and this emphasizes the need to combine evaluation of proximal arm control with the assessment of distal manual dexterity [26]. Coordinated hand and finger movements and versatile use, comprising functional opposites such as strength vs. precision, synergistic vs. individuated finger movements, or stability (e.g. grasp) vs. flexibility (e.g. object manipulation), are central features of manual dexterity. It is therefore unlikely that a single score may i) adequately assess the degree of dexterity which is multi-dimensional, and ii) differentiate between key elements of dexterity, whether in healthy subjects or after stroke. This is likely one of the reasons why there is currently no operational and widely accepted clinical definition of manual dexterity [27–29]. Knowledge is also lacking on how impaired dexterity affects activities of daily living. Nonetheless, some multidimensional aspects of dexterity are indirectly reflected by the combined use of outcome measures (in most of the studies, 72%) encompassing ICF body function as well as ICF activity items. This remains, however, an implicit and non quantitative approach to a differential description of key elements of dexterous upper limb function. A conceptually and operationally coherent characterization of manual dexterity and its key components may represent a critical future step not just for therapeutic intervention, but also for understanding of its neuronal underpinning [30, 31]. Another prerequisite for dexterous control is intact sensory function. However, only few studies used a specific measure of sensory function: only 0.6% of studies used the Nottingham Sensory Assessment. Nevertheless, some tests include a sensory assessment, e.g., in the FMT light touch and position sense is evaluated. The lack of specific tests for sensory or visual aspects is a major shortcoming across studies and recent findings show their importance for the recovery of upper limb function after stroke [32].

### Need for quantitative objective measures

The results of this study show limited and diverse use of many outcome measures (33 of the 48 different measures were used in less than 5% of studies, Fig 2). Many of these concern observer-based ordinal scales with questionable measurement properties. Nonetheless, even often-used measures have their limitations: these concern the psychometric properties (validity, reliability, objectivity and sensitivity) as well as the feasibility (cost-effectiveness, ease of administration, relevance for target population and for clinical and scientific question). For example, the measurement properties of the Ashworth scale are problematic: it is not considered a good measure of spasticity since the test is not objective, lacks a velocity component, and results in both false positives and false negatives, even in experienced raters [33]. The FMT, ARAT and MAS are ordinal scales where ceiling and floor effects are present [14, 34]. Timed grasping performance tests, such as the Box and Blocks Test (BBT) and Nine Hole Peg Test (NHPT), usually offer better reliability in mild-to-moderately than in severely affected stroke patients, and may suffer from poor sensitivity to change [35]. Similarly, the Wolf Motor Function Test (WMFT) and the Jebsen Taylor Hand Function Test (JTHFT), which are not affected by ceiling effects, are more suitable for patients with mild-to-moderate deficits [14]. More quantitative measures, such as Force control (FC) and Range of Motion (ROM), only inform on one aspect of the ICF body function domain and thus need to be combined with other measures for a broader evaluation of upper limb function. Finally, some measures are widely used in their country of origin but less so internationally, e.g., the Motor Assessment Scale (MAS) is highly used in Australia but not elsewhere.

Given these shortcomings, investigators generally select the most appropriate measures for the population studied and combine outcome measures. Investigators also increasingly opt for more quantitative upper limb measurement methods incorporating accelerometers and force sensors, which are becoming more frequent in stroke rehabilitation [36, 37]. In line with this observation we found an increasing trend for use of kinematic measures over time. Detailed movement and force analysis can offer insights into upper limb movement control in stroke and provide measures with enhanced sensitivity [38–40]. Given their higher sensitivity, kinematic and force control measurements can also provide more fine-grained measurement of sensorimotor changes in the upper limb. For example, using quantitative force control measures a recent study detected subtle upper limb impairments in patients with cervical spondylosis with mild or even absent neurological signs on conventional clinical testing [41]. Similarly, in patients with stroke, another study also detected impaired control of individual finger movements despite normal ARAT scores [42]. Quantitative assessments can also enhance diagnostic accuracy, for example, a new method of spasticity measurement detected a dose-dependent reduction of hand spasticity after injection of Botulinum toxin which remained undetected with conventional Ashworth rating scale [43]. New technology can also improve ecological validity, e.g., accelerometers can be used to monitor spontaneous use of the hand in the home environment which does not always correspond well with outcome measures [44]. Other studies have validated the clinical interest of these and other measures, for example by showing that some kinematic measures, such as reach movement time and smoothness during object lifting, are responsive to upper limb recovery after stroke [45] and more sensitive to change compared to conventional outcome measures [46]. Our results show a growing trend for the use of novel technology to improve the objective assessment of upper limb function after stroke [47, 48]. About 20% of the intervention studies in this review used assistive robots to train but also to measure upper limb function. Use of robots for measurement is promising since they offer more quantitative, objective and reliable measures than classical outcome measures. Robots may also allow measurement of aspects of sensory-motor integration difficult to assess clinically, such as visuospatial neglect or position sense [49, 50]. In addition, electrophysiological measures may be useful, especially given the importance of the corticospinal tract for recovery of manual dexterity [30, 51]. TMS measures, indicative of corticospinal integrity, can be meaningful for prediction of outcome and allow a better matching of severity across patients in clinical studies [52, 53]. However, a disadvantage with TMS and other advanced techniques (such as imaging) is that they are not readily feasible in typical rehabilitation settings since they require specialized equipment and particular skills for analysis and interpretation. Nonetheless, this is a growing field and future research aims at providing quantitative easy-to-use clinically applicable alternatives. Meanwhile clinical research studies could use standardized “core sets” of clinical tests, but further work is needed to identify and validate such core measures of upper limb function [14].

### Limitations

In this review we only included intervention studies. Data from longitudinal studies or from cross-sectional studies was not included. We limited inclusion to intervention studies in order to capture measures that clinicians and scientists consider appropriate to follow changes in upper limb recovery over time. Inclusion was also limited to studies from 2004 and after, with the goal of obtaining an up-to-date account of how upper limb function is currently measured in stroke studies.

We did not collect data about certain stroke-related factors that may have influenced the selection of outcome measures. Severity of paresis and time since stroke were not consistently reported. The terms acute, sub-acute and chronic were not included either, since they did not refer to consistent time windows across studies. We did not extract the type of setting, whether medical or research facility, since this information was not always available. Finally, we did not analyze whether the type of intervention influenced outcome measures selected.

### Conclusions

An increasing number of clinicians and scientists choose the Fugl-Meyer Test in post-stroke intervention studies to follow changes in upper limb function. Measurement of upper limb function after stroke is advancing and is more standardized than in earlier reports [54]. Furthermore, this study also provides evidence for the increased use of new technology, such as measures of movement kinematics. Although some National stroke care guidelines give recommendations on which outcome measures to use, consensus across countries is less established. International consensus could improve by establishing expert panels from different countries to decide on the most appropriate measures of upper limb function in stroke. This in turn would improve comparison across studies and feasibility of meta-analyses.

## Supporting Information

S1 Appendix Reference list of included studies. (PDF)

S1 Text PRISMA checklist. (PDF)
