# Supplementary material for: Upper Limb Outcome Measures Used in Stroke Rehabilitation Studies: A Systematic Literature Review
Source: PLoS One. 2016 May 6;11(5):e0154792. doi: 10.1371/journal.pone.0154792 (PMC4859525; doi:10.1371/journal.pone.0154792)
Supplement: S1 Appendix — (PDF) [file pone.0154792.s001.pdf]

1.
Abdollahi F, Case Lazarro ED, Listenberger M, Kenyon RV, Kovic M, Bogey RA, et al. Error augmentation enhancing arm recovery in individuals with chronic stroke: a randomized crossover design. Neurorehabil Neural Repair. 2014 Feb;28(2):120–8.

2.
Abdullah HA, Tarry C, Lambert C, Barreca S, Allen BO. Results of clinicians using a therapeutic robotic system in an inpatient stroke rehabilitation unit. J Neuroeng Rehabil. 2011;8:50.

3.
Ackerley SJ, Stinear CM, Barber PA, Byblow WD. Combining theta burst stimulation with training after subcortical stroke. Stroke. 2010 Jul;41(7):1568–72.

4.
Ackerley SJ, Stinear CM, Barber PA, Byblow WD. Priming sensorimotor cortex to enhance task-specific training after subcortical stroke. Clin Neurophysiol. 2014 Jul;125(7):1451–8.

5.
Acler M, Fiaschi A, Manganotti P. Long-term levodopa administration in chronic stroke patients. A clinical and neurophysiologic single-blind placebo-controlled cross-over pilot study. Restor Neurol Neurosci. 2009;27(4):277–83.

6.
Adamovich S, Fluet GG, Merians AS, Mathai A, Qiu Q. Recovery of hand function in virtual reality: Training hemiparetic hand and arm together or separately. Conf Proc IEEE Eng Med Biol Soc. 2008;2008:3475–8.

7.
Alberts JL, Butler AJ, Wolf SL. The effects of constraint-induced therapy on precision grip: a preliminary study. Neurorehabil Neural Repair. 2004 Dec;18(4):250–8.

8.
Alon G, Levitt AF, McCarthy PA. Functional electrical stimulation enhancement of upper extremity functional recovery during stroke rehabilitation: a pilot study. Neurorehabil Neural Repair. 2007 Jun;21(3):207–15.

9.
Alon G, Levitt AF, McCarthy PA. Functional electrical stimulation (FES) may modify the poor prognosis of stroke survivors with severe motor loss of the upper extremity: a preliminary study. Am J Phys Med Rehabil. 2008 Aug;87(8):627–36.

10.
Altenmüller E, Marco-Pallares J, Münte TF, Schneider S. Neural reorganization underlies improvement in stroke-induced motor dysfunction by music-supported therapy. Ann N Y Acad Sci. 2009 Jul;1169:395–405.

11.
Ameli M, Grefkes C, Kemper F, Riegg FP, Rehme AK, Karbe H, et al. Differential effects of high-frequency repetitive transcranial magnetic stimulation over ipsilesional primary motor cortex in cortical and subcortical middle cerebral artery stroke. Ann Neurol. 2009 Sep;66(3):298–309.

12.
Amengual JL, Rojo N, Veciana de Las Heras M, Marco-Pallarés J, Grau-Sánchez J, Schneider S, et al. Sensorimotor plasticity after music-supported therapy in chronic stroke patients revealed by transcranial magnetic stimulation. PLoS ONE. 2013;8(4):e61883.

13.
Ansari NN, Naghdi S, Fakhari Z, Radinmehr H, Hasson S. Dry needling for the treatment of poststroke muscle spasticity: a prospective case report. NeuroRehabilitation. 2015;36(1):61–5.

14.
Appel C, Perry L, Jones F. Testing a Protocol for a Randomized Controlled Trial of Therapeutic versus Placebo Shoulder Strapping as an Adjuvant Intervention Early after Stroke. Occup Ther Int. 2015 Jun;22(2):71–84.

15.
Arya KN, Pandian S. Effect of task-based mirror therapy on motor recovery of the upper extremity in chronic stroke patients: a pilot study. Top Stroke Rehabil. 2013 Jun;20(3):210–7.

16.
Askim T, Indredavik B, Håberg A. Internally and externally paced finger movements differ in reorganization after acute ischemic stroke. Arch Phys Med Rehabil. 2010 Oct;91(10):1529–36.

17.
Ausenda C, Carnovali M. Transfer of motor skill learning from the healthy hand to the paretic hand in stroke patients: a randomized controlled trial. Eur J Phys Rehabil Med. 2011 Sep;47(3):417–25.

18.
Au-Yeung SSY, Hui-Chan CWY. Electrical acupoint stimulation of the affected arm in acute stroke: a placebo-controlled randomized clinical trial. Clin Rehabil. 2014 Feb;28(2):149–58.

19.
Au-Yeung SSY, Wang J, Chen Y, Chua E. Transcranial direct current stimulation to primary motor area improves hand dexterity and selective attention in chronic stroke. Am J Phys Med Rehabil. 2014 Dec;93(12):1057–64.

20.
Bagce HF, Saleh S, Adamovich SV, Tunik E. Visuomotor gain distortion alters online motor performance and enhances primary motor cortex excitability in patients with stroke. Neuromodulation. 2012 Jul;15(4):361–6.

21.
Bai Y-L, Hu Y-S, Wu Y, Zhu Y-L, Zhang B, Jiang C-Y, et al. Long-term three-stage rehabilitation intervention alleviates spasticity of the elbows, fingers, and plantar flexors and improves activities of daily living in ischemic stroke patients: a randomized, controlled trial. Neuroreport. 2014 Sep 10;25(13):998–1005.

22.
Barker RN, Brauer SG, Barry BK, Gill TJ, Carson RG. Training-induced modifications of corticospinal reactivity in severely affected stroke survivors. Exp Brain Res. 2012 Aug;221(2):211–21.

23.
Barker RN, Brauer SG, Carson RG. Training of reaching in stroke survivors with severe and chronic upper limb paresis using a novel nonrobotic device: a randomized clinical trial. Stroke. 2008 Jun;39(6):1800–7.

24.
Barry JG, Ross SA, Woehrle J. Therapy incorporating a dynamic wrist-hand orthosis versus manual assistance in chronic stroke: a pilot study. J Neurol Phys Ther. 2012 Mar;36(1):17–24.

25.
Bartolo M, De Nunzio AM, Sebastiano F, Spicciato F, Tortola P, Nilsson J, et al. Arm weight support training improves functional motor outcome and movement smoothness after stroke. Funct Neurol. 2014 Mar;29(1):15–21.

26.
Barzel A, Liepert J, Haevernick K, Eisele M, Ketels G, Rijntjes M, et al. Comparison of two types of Constraint-Induced Movement Therapy in chronic stroke patients: A pilot study. Restor Neurol Neurosci. 2009;27(6):673–80.

27.
Basaran A, Emre U, Karadavut KI, Balbaloglu O, Bulmus N. Hand splinting for poststroke spasticity: a randomized controlled trial. Top Stroke Rehabil. 2012 Aug;19(4):329–37.

28.
Bensmail D, Sarfeld A-S, Ameli M, Fink GR, Nowak DA. Arbitrary visuomotor mapping in the grip-lift task: dissociation of performance deficits in right and left middle cerebral artery stroke. Neuroscience. 2012 May 17;210:128–36.

29.
Bensmail D, Robertson JVG, Fermanian C, Roby-Brami A. Botulinum toxin to treat upper-limb spasticity in hemiparetic patients: analysis of function and kinematics of reaching movements. Neurorehabil Neural Repair. 2010 Apr;24(3):273–81.

30.
Benvenuti F, Stuart M, Cappena V, Gabella S, Corsi S, Taviani A, et al. Community-based exercise for upper limb paresis: a controlled trial with telerehabilitation. Neurorehabil Neural Repair. 2014 Sep;28(7):611–20.

31.
Berends HI, Nijlant J, van Putten M, Movig KLL, IJzerman MJ. Single dose of fluoxetine increases muscle activation in chronic stroke patients. Clin Neuropharmacol. 2009 Feb;32(1):1–5.

32.
Bhakta BB, O’Connor RJ, Cozens JA. Associated reactions after stroke: a randomized controlled trial of the effect of botulinum toxin type A. J Rehabil Med. 2008 Jan;40(1):36–41.

33.
Bhatt E, Nagpal A, Greer KH, Grunewald TK, Steele JL, Wiemiller JW, et al. Effect of finger tracking combined with electrical stimulation on brain reorganization and hand function in subjects with stroke. Exp Brain Res. 2007 Oct;182(4):435–47.

34.

Blennerhassett J, Dite W. Additional task-related practice improves mobility and upper limb function early after stroke: a randomised controlled trial. Aust J Physiother. 2004;50(4):219–24.

35.
Blennerhassett JM, Avery RM, Carey LM. The test-retest reliability and responsiveness to change for the Hand Function Survey during stroke rehabilitation. Aust Occup Ther J. 2010 Dec;57(6):431–8.

36.
Blicher JU, Jakobsen J, Andersen G, Nielsen JF. Cortical excitability in chronic stroke and modulation by training: a TMS study. Neurorehabil Neural Repair. 2009 Jun;23(5):486–93.

37.
Boake C, Noser EA, Ro T, Baraniuk S, Gaber M, Johnson R, et al. Constraint-induced movement therapy during early stroke rehabilitation. Neurorehabil Neural Repair. 2007 Feb;21(1):14–24.

38.
Boggio PS, Alonso-Alonso M, Mansur CG, Rigonatti SP, Schlaug G, Pascual-Leone A, et al. Hand function improvement with low-frequency repetitive transcranial magnetic stimulation of the unaffected hemisphere in a severe case of stroke. Am J Phys Med Rehabil. 2006 Nov;85(11):927–30.

39.
Boggio PS, Nunes A, Rigonatti SP, Nitsche MA, Pascual-Leone A, Fregni F. Repeated sessions of noninvasive brain DC stimulation is associated with motor function improvement in stroke patients. Restor Neurol Neurosci. 2007;25(2):123–9.

40.
Bolognini N, Vallar G, Casati C, Latif LA, El-Nazer R, Williams J, et al. Neurophysiological and behavioral effects of tDCS combined with constraint-induced movement therapy in poststroke patients. Neurorehabil Neural Repair. 2011 Dec;25(9):819–29.

41.
Borstad AL, Bird T, Choi S, Goodman L, Schmalbrock P, Nichols-Larsen DS. Sensorimotor training and neural reorganization after stroke: a case series. J Neurol Phys Ther. 2013 Mar;37(1):27–36.

42.
Bowman MH, Taub E, Uswatte G, Delgado A, Bryson C, Morris DM, et al. A treatment for a chronic stroke patient with a plegic hand combining CI therapy with conventional rehabilitation procedures: case report. NeuroRehabilitation. 2006;21(2):167–76.

43.
Brashear A, McAfee AL, Kuhn ER, Fyffe J. Botulinum toxin type B in upper-limb poststroke spasticity: a double-blind, placebo-controlled trial. Arch Phys Med Rehabil. 2004 May;85(5):705–9.

44.
Brauer SG, Hayward KS, Carson RG, Cresswell AG, Barker RN. The efficacy of SMART Arm training early after stroke for stroke survivors with severe upper limb disability: a protocol for a randomised controlled trial. BMC Neurol. 2013;13:71.

45.
Broeren J, Rydmark M, Sunnerhagen KS. Virtual reality and haptics as a training device for movement rehabilitation after stroke: a single-case study. Arch Phys Med Rehabil. 2004 Aug;85(8):1247–50.

46.
Broetz D, Braun C, Weber C, Soekadar SR, Caria A, Birbaumer N. Combination of brain-computer interface training and goal-directed physical therapy in chronic stroke: a case report. Neurorehabil Neural Repair. 2010 Sep;24(7):674–9.

47.
Brogårdh C, Lexell J. A 1-year follow-up after shortened constraint-induced movement therapy with and without mitt poststroke. Arch Phys Med Rehabil. 2010 Mar;91(3):460–4.

48.
Brogårdh C, Sjölund BH. Constraint-induced movement therapy in patients with stroke: a pilot study on effects of small group training and of extended mitt use. Clin Rehabil. 2006 Mar;20(3):218–27.

49.
Brogårdh C, Vestling M, Sjölund BH. Shortened constraint-induced movement therapy in subacute stroke - no effect of using a restraint: a randomized controlled study with independent observers. J Rehabil Med. 2009 Mar;41(4):231–6.

50.
Brokaw EB, Black I, Holley RJ, Lum PS. Hand Spring Operated Movement Enhancer (HandSOME): a portable, passive hand exoskeleton for stroke rehabilitation. IEEE Trans Neural Syst Rehabil Eng. 2011 Aug;19(4):391–9.

51.
Brokaw EB, Nichols D, Holley RJ, Lum PS. Robotic therapy provides a stimulus for upper limb motor recovery after stroke that is complementary to and distinct from conventional therapy. Neurorehabil Neural Repair. 2014 May;28(4):367–76.

52.
Brown JA, Lutsep HL, Weinand M, Cramer SC. Motor cortex stimulation for the enhancement of recovery from stroke: a prospective, multicenter safety study. Neurosurgery. 2006 Mar;58(3):464–73.

53.
Brunner IC, Skouen JS, Strand LI. Is modified constraint-induced movement therapy more effective than bimanual training in improving arm motor function in the subacute phase post stroke? A randomized controlled trial. Clin Rehabil. 2012 Dec;26(12):1078–86.

54.
Burns A, Burridge J, Pickering R. Does the use of a constraint mitten to encourage use of the hemiplegic upper limb improve arm function in adults with subacute stroke? Clin Rehabil. 2007 Oct;21(10):895–904.

55.
Buschfort R, Brocke J, Hess A, Werner C, Waldner A, Hesse S. Arm studio to intensify the upper limb rehabilitation after stroke: concept, acceptance, utilization and preliminary clinical results. J Rehabil Med. 2010 Apr;42(4):310–4.

56.
Butler A, Blanton S, Rowe V, Wolf S. Attempting to improve function and quality of life using the FTM Protocol: case report. J Neurol Phys Ther. 2006 Sep;30(3):148–56.

57.
Butler AJ, Page SJ. Mental practice with motor imagery: evidence for motor recovery and cortical reorganization after stroke. Arch Phys Med Rehabil. 2006 Dec;87(12 Suppl 2):S2–11.

58.
Byl NN, Pitsch EA, Abrams GM. Functional outcomes can vary by dose: learning-based sensorimotor training for patients stable poststroke. Neurorehabil Neural Repair. 2008 Oct;22(5):494–504.

59.
Carey JR, Durfee WK, Bhatt E, Nagpal A, Weinstein SA, Anderson KM, et al. Comparison of finger tracking versus simple movement training via telerehabilitation to alter hand function and cortical reorganization after stroke. Neurorehabil Neural Repair. 2007 Jun;21(3):216–32.

60.
Carey L, Macdonell R, Matyas TA. SENSe: Study of the Effectiveness of Neurorehabilitation on Sensation: a randomized controlled trial. Neurorehabil Neural Repair. 2011 May;25(4):304–13.

61.
Carmeli E, Peleg S, Bartur G, Elbo E, Vatine J-J. HandTutor™ enhanced hand rehabilitation after stroke--a pilot study. Physiother Res Int. 2011 Dec;16(4):191–200.

62.
Cauraugh JH, Coombes SA, Lodha N, Naik SK, Summers JJ. Upper extremity improvements in chronic stroke: coupled bilateral load training. Restor Neurol Neurosci. 2009;27(1):17–25.

63.
Cauraugh JH, Kim S-B, Summers JJ. Chronic stroke longitudinal motor improvements: cumulative learning evidence found in the upper extremity. Cerebrovasc Dis. 2008;25(1-2):115–21.

64.
Cecatto RB. The effects of functional electrical stimulation on upper-extremity function and cortical plasticity in chronic stroke patients. Clin Neurophysiol. 2014 Aug;125(8):1709.

65.
Celnik P, Hummel F, Harris-Love M, Wolk R, Cohen LG. Somatosensory stimulation enhances the effects of training functional hand tasks in patients with chronic stroke. Arch Phys Med Rehabil. 2007 Nov;88(11):1369–76.

66.
Celnik P, Paik N-J, Vandermeeren Y, Dimyan M, Cohen LG. Effects of combined peripheral nerve stimulation and brain polarization on performance of a motor sequence task after chronic stroke. Stroke. 2009 May;40(5):1764–71.

67.
Celnik P, Webster B, Glasser DM, Cohen LG. Effects of action observation on physical training after stroke. Stroke. 2008 Jun;39(6):1814–20.

68.
Chae J, Harley MY, Hisel TZ, Corrigan CM, Demchak JA, Wong Y-T, et al. Intramuscular electrical stimulation for upper limb recovery in chronic hemiparesis: an exploratory randomized clinical trial. Neurorehabil Neural Repair. 2009 Aug;23(6):569–78.

69.
Chan MK-L, Tong RK-Y, Chung KY-K. Bilateral upper limb training with functional electric stimulation in patients with chronic stroke. Neurorehabil Neural Repair. 2009 May;23(4):357–65.

70.
Chang C-L, Munin MC, Skidmore ER, Niyonkuru C, Huber LM, Weber DJ. Effect of baseline spastic hemiparesis on recovery of upper-limb function following botulinum toxin type A injections and postinjection therapy. Arch Phys Med Rehabil. 2009 Sep;90(9):1462–8.

71.
Chang WH, Bang OY, Shin Y-I, Lee A, Pascual-Leone A, Kim Y-H. BDNF polymorphism and differential rTMS effects on motor recovery of stroke patients. Brain Stimul. 2014 Aug;7(4):553–8.

72.
Chang WH, Kim Y-H, Yoo W-K, Goo K-H, Park C-H, Kim ST, et al. rTMS with motor training modulates cortico-basal ganglia-thalamocortical circuits in stroke patients. Restor Neurol Neurosci. 2012;30(3):179–89.

73.
Chanubol R, Wongphaet P, Chavanich N, Werner C, Hesse S, Bardeleben A, et al. A randomized controlled trial of Cognitive Sensory Motor Training Therapy on the recovery of arm function in acute stroke patients. Clin Rehabil. 2012 Dec;26(12):1096–104.

74.
Chen J-C, Liang C-C, Shaw F-Z. Facilitation of sensory and motor recovery by thermal intervention for the hemiplegic upper limb in acute stroke patients: a single-blind randomized clinical trial. Stroke. 2005 Dec;36(12):2665–9.

75.
Childers MK, Brashear A, Jozefczyk P, Reding M, Alexander D, Good D, et al. Dose-dependent response to intramuscular botulinum toxin type A for upper-limb spasticity in patients after a stroke. Arch Phys Med Rehabil. 2004 Jul;85(7):1063–9.

76.
Cho S-Y, Kim M, Sun JJ, Jahng G-H, Kim HJ, Park S-U, et al. A comparison of brain activity between healthy subjects and stroke patients on fMRI by acupuncture stimulation. Chin J Integr Med. 2013 Apr;19(4):269–76.

77.
Cho YW, Jang SH, Lee ZI, Song JC, Lee HK, Lee HY. Effect and appropriate restriction period of constraint-induced movement therapy in hemiparetic patients with brain injury: a brief report. NeuroRehabilitation. 2005;20(2):71–4.

78.
Chouinard PA, Leonard G, Paus T. Changes in effective connectivity of the primary motor cortex in stroke patients after rehabilitative therapy. Exp Neurol. 2006 Oct;201(2):375–87.

79.
Church C, Price C, Pandyan AD, Huntley S, Curless R, Rodgers H. Randomized controlled trial to evaluate the effect of surface neuromuscular electrical stimulation to the shoulder after acute stroke. Stroke. 2006 Dec;37(12):2995–3001.

80.
Cirstea MC, Levin MF. Improvement of arm movement patterns and endpoint control depends on type of feedback during practice in stroke survivors. Neurorehabil Neural Repair. 2007 Oct;21(5):398–411.

81.
Combs SA, Finley MA, Henss M, Himmler S, Lapota K, Stillwell D. Effects of a repetitive gaming intervention on upper extremity impairments and function in persons with chronic stroke: a preliminary study. Disabil Rehabil. 2012;34(15):1291–8.

82.
Combs SA, Kelly SP, Barton R, Ivaska M, Nowak K. Effects of an intensive, task-specific rehabilitation program for individuals with chronic stroke: a case series. Disabil Rehabil. 2010;32(8):669–78.

83.
Conforto AB, Anjos SM, Saposnik G, Mello EA, Nagaya EM, Santos W, et al. Transcranial magnetic stimulation in mild to severe hemiparesis early after stroke: a proof of principle and novel approach to improve motor function. J Neurol. 2012 Jul;259(7):1399–405.

84.
Conforto AB, Cohen LG, Santos RL dos, Scaff M, Marie SKN. Effects of somatosensory stimulation on motor function in chronic cortico-subcortical strokes. J Neurol. 2007 Mar;254(3):333–9.

85.
Conforto AB, Ferreiro KN, Tomasi C, Santos RL dos, Moreira VL, Marie SKN, et al. Effects of somatosensory stimulation on motor function after subacute stroke. Neurorehabil Neural Repair. 2010 Apr;24(3):263–72.

86.
Connelly L, Jia Y, Toro ML, Stoykov ME, Kenyon RV, Kamper DG. A pneumatic glove and immersive virtual reality environment for hand rehabilitative training after stroke. IEEE Trans Neural Syst Rehabil Eng. 2010 Oct;18(5):551–9.

87.
Connelly L, Stoykov ME, Jia Y, Toro ML, Kenyon RV, Kamper DG. Use of a pneumatic glove for hand rehabilitation following stroke. Conf Proc IEEE Eng Med Biol Soc. 2009;2009:2434–7.

88.
Conrad MO, Scheidt RA, Schmit BD. Effects of wrist tendon vibration on targeted upper-arm movements in poststroke hemiparesis. Neurorehabil Neural Repair. 2011 Jan;25(1):61–70.

89.
Conrad MO, Scheidt RA, Schmit BD. Effects of wrist tendon vibration on arm tracking in people poststroke. J Neurophysiol. 2011 Sep;106(3):1480–8.

90.
Conti GE, Schepens SL. Changes in hemiplegic grasp following distributed repetitive intervention: a case series. Occup Ther Int. 2009;16(3-4):204–17.

91.
Coote S, Murphy B, Harwin W, Stokes E. The effect of the GENTLE/s robot-mediated therapy system on arm function after stroke. Clin Rehabil. 2008 May;22(5):395–405.

92.
Corti M, McGuirk TE, Wu SS, Patten C. Differential effects of power training versus functional task practice on compensation and restoration of arm function after stroke. Neurorehabil Neural Repair. 2012 Sep;26(7):842–54.

93.
Cousins E, Ward AB, Roffe C, Rimington LD, Pandyan AD. Quantitative measurement of poststroke spasticity and response to treatment with botulinum toxin: a 2-patient case report. Phys Ther. 2009 Jul;89(7):688–97.

94.
Craje C, van der Graaf C, Lem FC, Geurts ACH, Steenbergen B. Determining specificity of motor imagery training for upper limb improvement in chronic stroke patients: a training protocol and pilot results. Int J Rehabil Res. 2010 Dec;33(4):359–62.

95.
Dahl AE, Askim T, Stock R, Langørgen E, Lydersen S, Indredavik B. Short- and long-term outcome of constraint-induced movement therapy after stroke: a randomized controlled feasibility trial. Clin Rehabil. 2008 May;22(5):436–47.

96.
Daliri SS, Forogh B, Emami Razavi SZ, Ahadi T, Madjlesi F, Ansari NN. A single blind, clinical trial to investigate the effects of a single session extracorporeal shock wave therapy on wrist flexor spasticity after stroke. NeuroRehabilitation. 2015;36(1):67–72.

97.
Daly JJ, Cheng R, Rogers J, Litinas K, Hrovat K, Dohring M. Feasibility of a new application of noninvasive Brain Computer Interface (BCI): a case study of training for recovery of volitional motor control after stroke. J Neurol Phys Ther. 2009 Dec;33(4):203–11.

98.
Daly JJ, Hogan N, Perepezko EM, Krebs HI, Rogers JM, Goyal KS, et al. Response to upper-limb robotics and functional neuromuscular stimulation following stroke. J Rehabil Res Dev. 2005 Dec;42(6):723–36.

99.
da Silva Cameirão M, Bermúdez I Badia S, Duarte E, Verschure PFMJ. Virtual reality based rehabilitation speeds up functional recovery of the upper extremities after stroke: a randomized controlled pilot study in the acute phase of stroke using the rehabilitation gaming system. Restor Neurol Neurosci. 2011;29(5):287–98.

100.
Dean PJA, Seiss E, Sterr A. Motor planning in chronic upper-limb hemiparesis: evidence from movement-related potentials. PLoS ONE. 2012;7(10):e44558.

101.
de Araújo RC, Junior FL, Rocha DN, Sono TS, Pinotti M. Effects of intensive arm training with an electromechanical orthosis in chronic stroke patients: a preliminary study. Arch Phys Med Rehabil. 2011 Nov;92(11):1746–53.

102.
de Araújo RC, Rocha DN, Pitangui ACR, Pinotti M. The influence of dynamic orthosis training on upper extremity function after stroke: a pilot study. J Healthc Eng. 2014;5(1):55–66.

103.
de Jong LD, Dijkstra PU, Gerritsen J, Geurts ACH, Postema K. Combined arm stretch positioning and neuromuscular electrical stimulation during rehabilitation does not improve range of motion, shoulder pain or function in patients after stroke: a randomised trial. J Physiother. 2013 Dec;59(4):245–54.

104.
Dejong SL, Lang CE. Comparison of unilateral versus bilateral upper extremity task performance after stroke. Top Stroke Rehabil. 2012 Aug;19(4):294–305.

105.
DeJong SL, Schaefer SY, Lang CE. Need for speed: better movement quality during faster task performance after stroke. Neurorehabil Neural Repair. 2012 May;26(4):362–73.

106.
de Kroon JR, IJzerman MJ. Electrical stimulation of the upper extremity in stroke: cyclic versus EMG-triggered stimulation. Clin Rehabil. 2008 Aug;22(8):690–7.

107.
de Kroon JR, IJzerman MJ, Lankhorst GJ, Zilvold G. Electrical stimulation of the upper limb in stroke: stimulation of the extensors of the hand vs. alternate stimulation of flexors and extensors. Am J Phys Med Rehabil. 2004 Aug;83(8):592–600.

108.
Desrosiers J, Bourbonnais D, Corriveau H, Gosselin S, Bravo G. Effectiveness of unilateral and symmetrical bilateral task training for arm during the subacute phase after stroke: a randomized controlled trial. Clin Rehabil. 2005 Sep;19(6):581–93.

109.
Dettmers C, Benz M, Liepert J, Rockstroh B. Motor imagery in stroke patients, or plegic patients with spinal cord or peripheral diseases. Acta Neurol Scand. 2012 Oct;126(4):238–47.

110.
Dettmers C, Teske U, Hamzei F, Uswatte G, Taub E, Weiller C. Distributed form of constraint-induced movement therapy improves functional outcome and quality of life after stroke. Arch Phys Med Rehabil. 2005 Feb;86(2):204–9.

111.
Dijkerman HC, Ietswaart M, Johnston M, MacWalter RS. Does motor imagery training improve hand function in chronic stroke patients? A pilot study. Clin Rehabil. 2004 Aug;18(5):538–49.

112.

Diserens K, Ruegg D, Kleiser R, Hyde S, Perret N, Vuadens P, et al. Effect of repetitive arm cycling following botulinum toxin injection for poststroke spasticity: evidence from FMRI. Neurorehabil Neural Repair. 2010 Oct;24(8):753–62.

113.
Doğan-Aslan M, Nakipoğlu-Yüzer GF, Doğan A, Karabay I, Özgirgin N. The effect of electromyographic biofeedback treatment in improving upper extremity functioning of patients with hemiplegic stroke. J Stroke Cerebrovasc Dis. 2012 Apr;21(3):187–92.

114.
Dong Y, Winstein CJ, Albistegui-DuBois R, Dobkin BH. Evolution of FMRI activation in the perilesional primary motor cortex and cerebellum with rehabilitation training-related motor gains after stroke: a pilot study. Neurorehabil Neural Repair. 2007 Oct;21(5):412–28.

115.
Donoso Brown EV, McCoy SW, Fechko AS, Price R, Gilbertson T, Moritz CT. Preliminary investigation of an electromyography-controlled video game as a home program for persons in the chronic phase of stroke recovery. Arch Phys Med Rehabil. 2014 Aug;95(8):1461–9.

116.
Dorsch S, Ada L, Canning CG. EMG-triggered electrical stimulation is a feasible intervention to apply to multiple arm muscles in people early after stroke, but does not improve strength and activity more than usual therapy: a randomized feasibility trial. Clin Rehabil. 2014 May;28(5):482–90.

117.
Doucet BM, Griffin L. Variable stimulation patterns for poststroke hemiplegia. Muscle Nerve. 2009 Jan;39(1):54–62.

118.
Doucet BM, Griffin L. High-versus low-frequency stimulation effects on fine motor control in chronic hemiplegia: a pilot study. Top Stroke Rehabil. 2013 Aug;20(4):299–307.

119.
Doucet BM, Mettler JA. Effects of a dynamic progressive orthotic intervention for chronic hemiplegia: a case series. J Hand Ther. 2013 Jun;26(2):139–46; quiz 147.

120.
Dromerick AW, Lang CE, Birkenmeier RL, Wagner JM, Miller JP, Videen TO, et al. Very Early Constraint-Induced Movement during Stroke Rehabilitation (VECTORS): A single-center RCT. Neurology. 2009 Jul 21;73(3):195–201.

121.
Duff M, Chen Y, Attygalle S, Herman J, Sundaram H, Qian G, et al. An adaptive mixed reality training system for stroke rehabilitation. IEEE Trans Neural Syst Rehabil Eng. 2010 Oct;18(5):531–41.

122.
Dunning K, Berberich A, Albers B, Mortellite K, Levine PG, Hill Hermann VA, et al. A four-week, task-specific neuroprosthesis program for a person with no active wrist or finger movement because of chronic stroke. Phys Ther. 2008 Mar;88(3):397–405.

123.

Durham KF, Sackley CM, Wright CC, Wing AM, Edwards MG, van Vliet P. Attentional focus of feedback for improving performance of reach-to-grasp after stroke: a randomised crossover study. Physiotherapy. 2014 Jun;100(2):108–15.

124.
Edwards DJ, Krebs HI, Rykman A, Zipse J, Thickbroom GW, Mastaglia FL, et al. Raised corticomotor excitability of M1 forearm area following anodal tDCS is sustained during robotic wrist therapy in chronic stroke. Restor Neurol Neurosci. 2009;27(3):199–207.

125.
Ellis MD, Sukal-Moulton T, Dewald JPA. Progressive shoulder abduction loading is a crucial element of arm rehabilitation in chronic stroke. Neurorehabil Neural Repair. 2009 Oct;23(8):862–9.

126.
Elovic EP, Brashear A, Kaelin D, Liu J, Millis SR, Barron R, et al. Repeated treatments with botulinum toxin type a produce sustained decreases in the limitations associated with focal upper-limb poststroke spasticity for caregivers and patients. Arch Phys Med Rehabil. 2008 May;89(5):799–806.

127.
Emara TH, Moustafa RR, Elnahas NM, Elganzoury AM, Abdo TA, Mohamed SA, et al. Repetitive transcranial magnetic stimulation at 1Hz and 5Hz produces sustained improvement in motor function and disability after ischaemic stroke. Eur J Neurol. 2010 Sep;17(9):1203–9.

128.
Etoh S, Noma T, Ikeda K, Jonoshita Y, Ogata A, Matsumoto S, et al. Effects of repetitive trascranial magnetic stimulation on repetitive facilitation exercises of the hemiplegic hand in chronic stroke patients. J Rehabil Med. 2013 Sep;45(9):843–7.

129.
Fabbrini S, Casati G, Bonaiuti D. Is CIMT a rehabilitative practice for everyone? Predictive factors and feasibility. Eur J Phys Rehabil Med. 2014 Oct;50(5):505–14.

130.
Facca S, Louis P, Isner M-E, Gault D, Allieu Y, Liverneaux P. Braun’s flexor tendons transfer in disabled hands by central nervous system lesions. Orthop Traumatol Surg Res. 2010 Oct;96(6):656–61.

131.
Farrell JF, Hoffman HB, Snyder JL, Giuliani CA, Bohannon RW. Orthotic aided training of the paretic upper limb in chronic stroke: results of a phase 1 trial. NeuroRehabilitation. 2007;22(2):99–103.

132.
Fischer HC, Stubblefield K, Kline T, Luo X, Kenyon RV, Kamper DG. Hand rehabilitation following stroke: a pilot study of assisted finger extension training in a virtual environment. Top Stroke Rehabil. 2007 Feb;14(1):1–12.

133.
Flinn NA, Smith JL, Tripp CJ, White MW. Effects of robotic-aided rehabilitation on recovery of upper extremity function in chronic stroke: a single case study. Occup Ther Int. 2009;16(3-4):232–43.

134.
Floel A, Hummel F, Breitenstein C, Knecht S, Cohen LG. Dopaminergic effects on encoding of a motor memory in chronic stroke. Neurology. 2005 Aug 9;65(3):472–4.

135.
Floel A, Hummel F, Duque J, Knecht S, Cohen LG. Influence of somatosensory input on interhemispheric interactions in patients with chronic stroke. Neurorehabil Neural Repair. 2008 Oct;22(5):477–85.

136.
Fluet GG, Merians AS, Qiu Q, Lafond I, Saleh S, Ruano V, et al. Robots integrated with virtual reality simulations for customized motor training in a person with upper extremity hemiparesis: a case study. J Neurol Phys Ther. 2012 Jun;36(2):79–86.

137.
Fong KN, Lo PC, Yu YS, Cheuk CK, Tsang TH, Po AS, et al. Effects of sensory cueing on voluntary arm use for patients with chronic stroke: a preliminary study. Arch Phys Med Rehabil. 2011 Jan;92(1):15–23.

138.
Franceschini M, Agosti M, Cantagallo A, Sale P, Mancuso M, Buccino G. Mirror neurons: action observation treatment as a tool in stroke rehabilitation. Eur J Phys Rehabil Med. 2010 Dec;46(4):517–23.

139.
Fregni F, Boggio PS, Valle AC, Rocha RR, Duarte J, Ferreira MJL, et al. A sham-controlled trial of a 5-day course of repetitive transcranial magnetic stimulation of the unaffected hemisphere in stroke patients. Stroke. 2006 Aug;37(8):2115–22.

140.
Frick EM, Alberts JL. Combined use of repetitive task practice and an assistive robotic device in a patient with subacute stroke. Phys Ther. 2006 Oct;86(10):1378–86.

141.
Friedman N, Chan V, Reinkensmeyer AN, Beroukhim A, Zambrano GJ, Bachman M, et al. Retraining and assessing hand movement after stroke using the MusicGlove: comparison with conventional hand therapy and isometric grip training. J Neuroeng Rehabil. 2014;11:76.

142.
Fritz SL, Light KE, Clifford SN, Patterson TS, Behrman AL, Davis SB. Descriptive characteristics as potential predictors of outcomes following constraint-induced movement therapy for people after stroke. Phys Ther. 2006 Jun;86(6):825–32.

143.
Fritz SL, Light KE, Patterson TS, Behrman AL, Davis SB. Active finger extension predicts outcomes after constraint-induced movement therapy for individuals with hemiparesis after stroke. Stroke. 2005 Jun;36(6):1172–7.

144.
Fusco A, Assenza F, Iosa M, Izzo S, Altavilla R, Paolucci S, et al. The ineffective role of cathodal tDCS in enhancing the functional motor outcomes in early phase of stroke rehabilitation: an experimental trial. Biomed Res Int. 2014;2014:547290.

145.
Fusco A, Iosa M, Venturiero V, De Angelis D, Morone G, Maglione L, et al. After vs. priming effects of anodal transcranial direct current stimulation on upper extremity motor recovery in patients with subacute stroke. Restor Neurol Neurosci. 2014;32(2):301–12.

146.
Gladstone DJ, Danells CJ, Armesto A, McIlroy WE, Staines WR, Graham SJ, et al. Physiotherapy coupled with dextroamphetamine for rehabilitation after hemiparetic stroke: a randomized, double-blind, placebo-controlled trial. Stroke. 2006 Jan;37(1):179–85.

147.
Godfrey SB, Holley RJ, Lum PS. Comparison of Tone compensation and Spring assistance for hand rehabilitation in HEXORR. Conf Proc IEEE Eng Med Biol Soc. 2011;2011:8535–8.

148.
Godfrey SB, Schabowsky CN, Holley RJ, Lum PS. Hand function recovery in chronic stroke with HEXORR robotic training: A case series. Conf Proc IEEE Eng Med Biol Soc. 2010;2010:4485–8.

149.
Grefkes C, Nowak DA, Wang LE, Dafotakis M, Eickhoff SB, Fink GR. Modulating cortical connectivity in stroke patients by rTMS assessed with fMRI and dynamic causal modeling. Neuroimage. 2010 Mar;50(1):233–42.

150.
Gritsenko V, Prochazka A. A functional electric stimulation-assisted exercise therapy system for hemiplegic hand function. Arch Phys Med Rehabil. 2004 Jun;85(6):881–5.

151.
Guttman A, Burstin A, Brown R, Bril S, Dickstein R. Motor imagery practice for improving sit to stand and reaching to grasp in individuals with poststroke hemiparesis. Top Stroke Rehabil. 2012 Aug;19(4):306–19.

152.
Hakim RM, Kelly SJ, Grant-Beuttler M, Healy B, Krempasky J, Moore S. Case report: a modified constraint-induced therapy (mCIT) program for the upper extremity of a person with chronic stroke. Physiother Theory Pract. 2005 Dec;21(4):243–56.

153.
Hammer AM, Lindmark B. Effects of forced use on arm function in the subacute phase after stroke: a randomized, clinical pilot study. Phys Ther. 2009 Jun;89(6):526–39.

154.
Hara Y, Obayashi S, Tsujiuchi K, Muraoka Y. The effects of electromyography-controlled functional electrical stimulation on upper extremity function and cortical perfusion in stroke patients. Clin Neurophysiol. 2013 Oct;124(10):2008–15.

155.
Hara Y, Ogawa S, Muraoka Y. Hybrid power-assisted functional electrical stimulation to improve hemiparetic upper-extremity function. Am J Phys Med Rehabil. 2006 Dec;85(12):977–85.

156.

Harris JE, Eng JJ, Miller WC, Dawson AS. A self-administered Graded Repetitive Arm Supplementary Program (GRASP) improves arm function during inpatient stroke rehabilitation: a multi-site randomized controlled trial. Stroke. 2009 Jun;40(6):2123–8.

157. Harris JE, Eng JJ, Miller WC, Dawson AS. The role of caregiver involvement in upper-limb treatment in individuals with subacute stroke. Phys Ther. 2010 Sep;90(9):1302–10.

158. Harris-Love ML, McCombe Waller S, Whitall J. Exploiting interlimb coupling to improve paretic arm reaching performance in people with chronic stroke. Arch Phys Med Rehabil. 2005 Nov;86(11):2131–7.

159. Harvey L, de Jong I, Goehl G, Mardwedel S. Twelve weeks of nightly stretch does not reduce thumb web-space contractures in people with a neurological condition: a randomised controlled trial. Aust J Physiother. 2006;52(4):251–8.

160. Hayward KS, Barker RN, Brauer SG, Lloyd D, Horsley SA, Carson RG. SMART Arm with outcome-triggered electrical stimulation: a pilot randomized clinical trial. Top Stroke Rehabil. 2013 Aug;20(4):289–98.

161. Hemmen B, Seelen H a. M. Effects of movement imagery and electromyography-triggered feedback on arm hand function in stroke patients in the subacute phase. Clin Rehabil. 2007 Jul;21(7):587–94.

162. Hesse S, Mach H, Fröhlich S, Behrend S, Werner C, Melzer I. An early botulinum toxin A treatment in subacute stroke patients may prevent a disabling finger flexor stiffness six months later: a randomized controlled trial. Clin Rehabil. 2012 Mar;26(3):237–45.

163. Hesse S, Werner C, Pohl M, Rueckriem S, Mehrholz J, Lingnau ML. Computerized arm training improves the motor control of the severely affected arm after stroke: a single-blinded randomized trial in two centers. Stroke. 2005 Sep;36(9):1960–6.

164. Hesse S, Werner C, Schonhardt EM, Bardeleben A, Jenrich W, Kirker SGB. Combined transcranial direct current stimulation and robot-assisted arm training in subacute stroke patients: a pilot study. Restor Neurol Neurosci. 2007;25(1):9–15.

165. Hesse S, Kuhlmann H, Wilk J, Tomelleri C, Kirker SGB. A new electromechanical trainer for sensorimotor rehabilitation of paralysed fingers: a case series in chronic and acute stroke patients. J Neuroeng Rehabil. 2008;5:21.

166. Hesse S, Waldner A, Mehrholz J, Tomelleri C, Pohl M, Werner C. Combined transcranial direct current stimulation and robot-assisted arm training in subacute stroke patients: an exploratory, randomized multicenter trial. Neurorehabil Neural Repair. 2011 Dec;25(9):838–46.

167.
Hicks CM, Kluding P. Modification of constraint induced movement therapy in the home health setting for a subject with chronic hemiparesis after stroke. J Geriatr Phys Ther. 2008;31(3):113–9.

168.
Higgins J, Salbach NM, Wood-Dauphinee S, Richards CL, Côté R, Mayo NE. The effect of a task-oriented intervention on arm function in people with stroke: a randomized controlled trial. Clin Rehabil. 2006 Apr;20(4):296–310.

169.
Hijmans JM, Hale LA, Satherley JA, McMillan NJ, King MJ. Bilateral upper-limb rehabilitation after stroke using a movement-based game controller. J Rehabil Res Dev. 2011;48(8):1005–13.

170.
Hoffmann G, Schmit BD, Kahn JH, Kamper DG. Effect of sensory feedback from the proximal upper limb on voluntary isometric finger flexion and extension in hemiparetic stroke subjects. J Neurophysiol. 2011 Nov;106(5):2546–56.

171.
Holden MK, Dyar TA, Dayan-Cimadoro L. Telerehabilitation using a virtual environment improves upper extremity function in patients with stroke. IEEE Trans Neural Syst Rehabil Eng. 2007 Mar;15(1):36–42.

172.
Horsley SA, Herbert RD, Ada L. Four weeks of daily stretch has little or no effect on wrist contracture after stroke: a randomised controlled trial. Aust J Physiother. 2007;53(4):239–45.

173.
Hosomi M, Koyama T, Takebayashi T, Terayama S, Kodama N, Matsumoto K, et al. A modified method for constraint-induced movement therapy: a supervised self-training protocol. J Stroke Cerebrovasc Dis. 2012 Nov;21(8):767–75.

174.
Hovington CL, Brouwer B. Guided motor imagery in healthy adults and stroke: does strategy matter? Neurorehabil Neural Repair. 2010 Dec;24(9):851–7.

175.
Hsieh Y, Lin K, Wu C, Lien H, Chen J, Chen C, et al. Predicting clinically significant changes in motor and functional outcomes after robot-assisted stroke rehabilitation. Arch Phys Med Rehabil. 2014 Feb;95(2):316–21.

176.
Hsu H-Y, Lin C-F, Su F-C, Kuo H-T, Chiu H-Y, Kuo L-C. Clinical application of computerized evaluation and re-education biofeedback prototype for sensorimotor control of the hand in stroke patients. J Neuroeng Rehabil. 2012;9:26.

177.
Hsu S-S, Hu M-H, Wang Y-H, Yip P-K, Chiu J-W, Hsieh C-L. Dose-response relation between neuromuscular electrical stimulation and upper-extremity function in patients with stroke. Stroke. 2010 Apr;41(4):821–4.

178.
Hsu Y-F, Huang Y-Z, Lin Y-Y, Tang C-W, Liao K-K, Lee P-L, et al. Intermittent theta burst stimulation over ipsilesional primary motor cortex of subacute ischemic stroke patients: a pilot study. Brain Stimul. 2013 Mar;6(2):166–74.

179.
Hu XL, Tong KY, Li R, Chen M, Xue JJ, Ho SK, et al. Effectiveness of functional electrical stimulation (FES)-robot assisted wrist training on persons after stroke. Conf Proc IEEE Eng Med Biol Soc. 2010;2010:5819–22.

180.
Hu XL, Tong KY, Li R, Xue JJ, Ho SK, Chen P. The effects of electromechanical wrist robot assistive system with neuromuscular electrical stimulation for stroke rehabilitation. J Electromyogr Kinesiol. 2012 Jun;22(3):431–9.

181.
Huang M, Harvey RL, Stoykov ME, Ruland S, Weinand M, Lowry D, et al. Cortical stimulation for upper limb recovery following ischemic stroke: a small phase II pilot study of a fully implanted stimulator. Top Stroke Rehabil. 2008 Apr;15(2):160–72.

182.
Hughes AM, Freeman CT, Burridge JH, Chappell PH, Lewin PL, Rogers E. Feasibility of iterative learning control mediated by functional electrical stimulation for reaching after stroke. Neurorehabil Neural Repair. 2009 Aug;23(6):559–68.

183.
Huijgen BCH, Vollenbroek-Hutten MMR, Zampolini M, Opisso E, Bernabeu M, Van Nieuwenhoven J, et al. Feasibility of a home-based telerehabilitation system compared to usual care: arm/hand function in patients with stroke, traumatic brain injury and multiple sclerosis. J Telemed Telecare. 2008;14(5):249–56.

184.
Hummel F, Celnik P, Giraux P, Floel A, Wu W-H, Gerloff C, et al. Effects of non-invasive cortical stimulation on skilled motor function in chronic stroke. Brain. 2005 Mar;128(Pt 3):490–9.

185.
Hummel F, Cohen LG. Improvement of motor function with noninvasive cortical stimulation in a patient with chronic stroke. Neurorehabil Neural Repair. 2005 Mar;19(1):14–9.

186.
Hummel FC, Voller B, Celnik P, Floel A, Giraux P, Gerloff C, et al. Effects of brain polarization on reaction times and pinch force in chronic stroke. BMC Neurosci. 2006;7:73.

187.
Ibrahimpasic T, Ghossein R, Carlson DL, Chernichenko N, Nixon I, Palmer FL, et al. Poorly differentiated thyroid carcinoma presenting with gross extrathyroidal extension: 1986-2009 Memorial Sloan-Kettering Cancer Center experience. Thyroid. 2013 Aug;23(8):997–1002.

188.
Ietswaart M, Johnston M, Dijkerman HC, Joice S, Scott CL, MacWalter RS, et al. Mental practice with motor imagery in stroke recovery: randomized controlled trial of efficacy. Brain. 2011 May;134(Pt 5):1373–86.

189.
Ikuno K, Kawaguchi S, Kitabeppu S, Kitaura M, Tokuhisa K, Morimoto S, et al. Effects of peripheral sensory nerve stimulation plus task-oriented training on upper extremity function in patients with subacute stroke: a pilot randomized crossover trial. Clin Rehabil. 2012 Nov;26(11):999–1009.

190.
Inobe J, Kato T. Effectiveness of finger-equipped electrode (FEE)-triggered electrical stimulation improving chronic stroke patients with severe hemiplegia. Brain Inj. 2013;27(1):114–9.

191.
Iwamuro BT, Fischer HC, Kamper DG. A pilot study to assess use of passive extension bias to facilitate finger movement for repetitive task practice after stroke. Top Stroke Rehabil. 2011 Aug;18(4):308–15.

192.
Izumi S-I, Kondo T, Shindo K. Transcranial magnetic stimulation synchronized with maximal movement effort of the hemiplegic hand after stroke: a double-blinded controlled pilot study. J Rehabil Med. 2008 Jan;40(1):49–54.

193.
Jahangir AW, Tan HJ, Norlinah MI, Nafisah WY, Ramesh S, Hamidon BB, et al. Intramuscular injection of botulinum toxin for the treatment of wrist and finger spasticity after stroke. Med J Malaysia. 2007 Oct;62(4):319–22.

194.
Jang SH. Contra-lesional somatosensory cortex activity and somatosensory recovery in two stroke patients. J Rehabil Med. 2011 Feb;43(3):268–70.

195.
Jeon H, Woo Y-K, Yi C, Kwon O, Jung M, Lee Y, et al. Effect of intensive training with a spring-assisted hand orthosis on movement smoothness in upper extremity following stroke: a pilot clinical trial. Top Stroke Rehabil. 2012 Aug;19(4):320–8.

196.
Jo HM, Song J, Jang SH. Improvements in spasticity and motor function using a static stretching device for people with chronic hemiparesis following stroke. NeuroRehabilitation. 2013;32(2):369–75.

197.
Jordan K, Sampson M, King M. Gravity-supported exercise with computer gaming improves arm function in chronic stroke. Arch Phys Med Rehabil. 2014 Aug;95(8):1484–9.

198.
Jung YJ, Hong JH, Kwon HG, Song J-C, Kim C, Park S, et al. The effect of a stretching device on hand spasticity in chronic hemiparetic stroke patients. NeuroRehabilitation. 2011;29(1):53–9.

199.
Kakuda W, Abo M, Kobayashi K, Momosaki R, Yokoi A, Fukuda A, et al. Low-frequency repetitive transcranial magnetic stimulation and intensive occupational therapy for poststroke

patients with upper limb hemiparesis: preliminary study of a 15-day protocol. Int J Rehabil Res. 2010 Dec;33(4):339–45.

200.
Kakuda W, Abo M, Kobayashi K, Momosaki R, Yokoi A, Fukuda A, et al. Anti-spastic effect of low-frequency rTMS applied with occupational therapy in post-stroke patients with upper limb hemiparesis. Brain Inj. 2011;25(5):496–502.

201.
Kakuda W, Abo M, Kobayashi K, Takagishi T, Momosaki R, Yokoi A, et al. Baseline severity of upper limb hemiparesis influences the outcome of low-frequency rTMS combined with intensive occupational therapy in patients who have had a stroke. PM R. 2011 Jun;3(6):516–22; quiz 522.

202.
Kang HS, Sok SR, Kang JS. Effects of Meridian acupressure for stroke patients in Korea. J Clin Nurs. 2009 Aug;18(15):2145–52.

203.
Kang N, Cauraugh JH. Force control improvements in chronic stroke: bimanual coordination and motor synergy evidence after coupled bimanual movement training. Exp Brain Res. 2014 Feb;232(2):503–13.

204.
Kang N, Cauraugh JH. Force frequency structure below 1 Hz in chronic stroke: paretic arm control. Brain Res. 2014 Jun 20;1569:32–40.

205.
Kang N, Idica J, Amitoj B, Cauraugh JH. Motor recovery patterns in arm muscles: coupled bilateral training and neuromuscular stimulation. J Neuroeng Rehabil. 2014;11:57.

206.
Kaňovský P, Slawek J, Denes Z, Platz T, Comes G, Grafe S, et al. Efficacy and safety of treatment with incobotulinum toxin A (botulinum neurotoxin type A free from complexing proteins; NT 201) in post-stroke upper limb spasticity. J Rehabil Med. 2011 May;43(6):486–92.

207.
Kawahira K, Shimodozono M, Etoh S, Kamada K, Noma T, Tanaka N. Effects of intensive repetition of a new facilitation technique on motor functional recovery of the hemiplegic upper limb and hand. Brain Inj. 2010;24(10):1202–13.

208.
Khan CM, Oesch PR, Gamper UN, Kool JP, Beer S. Potential effectiveness of three different treatment approaches to improve minimal to moderate arm and hand function after stroke--a pilot randomized clinical trial. Clin Rehabil. 2011 Nov;25(11):1032–41.

209.
Kim D-Y, Lim J-Y, Kang EK, You DS, Oh M-K, Oh B-M, et al. Effect of transcranial direct current stimulation on motor recovery in patients with subacute stroke. Am J Phys Med Rehabil. 2010 Nov;89(11):879–86.

210.

Kim DG, Cho YW, Hong JH, Song JC, Chung H-A, Bai D, et al. Effect of constraint-induced movement therapy with modified opposition restriction orthosis in chronic hemiparetic patients with stroke. NeuroRehabilitation. 2008;23(3):239–44.

211.
Kim H, Lee G, Song C. Effect of functional electrical stimulation with mirror therapy on upper extremity motor function in poststroke patients. J Stroke Cerebrovasc Dis. 2014 Apr;23(4):655–61.

212.
Kim NH, Wininger M, Craelius W. Training grip control with a Fitts' paradigm: a pilot study in chronic stroke. J Hand Ther. 2010 Mar;23(1):63–71; quiz 72.

213.
Kim Y-H, You SH, Ko M-H, Park J-W, Lee KH, Jang SH, et al. Repetitive transcranial magnetic stimulation-induced corticomotor excitability and associated motor skill acquisition in chronic stroke. Stroke. 2006 Jun;37(6):1471–6.

214.
Kimberley TJ, Lewis SM, Auerbach EJ, Dorsey LL, Lojovich JM, Carey JR. Electrical stimulation driving functional improvements and cortical changes in subjects with stroke. Exp Brain Res. 2004 Feb;154(4):450–60.

215.
Kitago T, Liang J, Huang VS, Hayes S, Simon P, Tenteromano L, et al. Improvement after constraint-induced movement therapy: recovery of normal motor control or task-specific compensation? Neurorehabil Neural Repair. 2013 Feb;27(2):99–109.

216.
Klaiput A, Kitisomprayoonkul W. Increased pinch strength in acute and subacute stroke patients after simultaneous median and ulnar sensory stimulation. Neurorehabil Neural Repair. 2009 May;23(4):351–6.

217.
Knutson JS, Chae J, Hart RL, Keith MW, Hoyen HA, Harley MY, et al. Implanted neuroprosthesis for assisting arm and hand function after stroke: a case study. J Rehabil Res Dev. 2012;49(10):1505–16.

218.
Knutson JS, Harley MY, Hisel TZ, Chae J. Improving hand function in stroke survivors: a pilot study of contralaterally controlled functional electric stimulation in chronic hemiplegia. Arch Phys Med Rehabil. 2007 Apr;88(4):513–20.

219.
Knutson JS, Harley MY, Hisel TZ, Hogan SD, Maloney MM, Chae J. Contralaterally controlled functional electrical stimulation for upper extremity hemiplegia: an early-phase randomized clinical trial in subacute stroke patients. Neurorehabil Neural Repair. 2012 Apr;26(3):239–46.

220.
Knutson JS, Harley MY, Hisel TZ, Makowski NS, Chae J. Contralaterally controlled functional electrical stimulation for recovery of elbow extension and hand opening after stroke: a pilot case series study. Am J Phys Med Rehabil. 2014 Jun;93(6):528–39.

221.
Koganemaru S, Mima T, Thabit MN, Ikkaku T, Shimada K, Kanematsu M, et al. Recovery of upper-limb function due to enhanced use-dependent plasticity in chronic stroke patients. Brain. 2010 Nov;133(11):3373–84.

222.
Kojima K, Ikuno K, Morii Y, Tokuhisa K, Morimoto S, Shomoto K. Feasibility study of a combined treatment of electromyography-triggered neuromuscular stimulation and mirror therapy in stroke patients: a randomized crossover trial. NeuroRehabilitation. 2014;34(2):235–44.

223.
Kondziolka D, Steinberg GK, Wechsler L, Meltzer CC, Elder E, Gebel J, et al. Neurotransplantation for patients with subcortical motor stroke: a phase 2 randomized trial. J Neurosurg. 2005 Jul;103(1):38–45.

224.
Könönen M, Tarkka IM, Niskanen E, Pihlajamäki M, Mervaala E, Pitkänen K, et al. Functional MRI and motor behavioral changes obtained with constraint-induced movement therapy in chronic stroke. Eur J Neurol. 2012 Apr;19(4):578–86.

225.
Könönen M, Kuikka JT, Husso-Saastamoinen M, Vanninen E, Vanninen R, Soimakallio S, et al. Increased perfusion in motor areas after constraint-induced movement therapy in chronic stroke: a single-photon emission computerized tomography study. J Cereb Blood Flow Metab. 2005 Dec;25(12):1668–74.

226.
Kowalczewski J, Gritsenko V, Ashworth N, Ellaway P, Prochazka A. Upper-extremity functional electric stimulation-assisted exercises on a workstation in the subacute phase of stroke recovery. Arch Phys Med Rehabil. 2007 Jul;88(7):833–9.

227.
Koyama S, Tanabe S, Warashina H, Kaneko T, Sakurai H, Kanada Y, et al. NMES with rTMS for moderate to severe dysfunction after stroke. NeuroRehabilitation. 2014;35(3):363–8.

228.
Krabben T, Prange GB, Molier BI, Stienen AHA, Jannink MJA, Buurke JH, et al. Influence of gravity compensation training on synergistic movement patterns of the upper extremity after stroke, a pilot study. J Neuroeng Rehabil. 2012;9:44.

229.
Krebs HI, Mernoff S, Fasoli SE, Hughes R, Stein J, Hogan N. A comparison of functional and impairment-based robotic training in severe to moderate chronic stroke: a pilot study. NeuroRehabilitation. 2008;23(1):81–7.

230.
Krukowska J, Swiątek E, Sienkiewicz M, Czernicki J. Influence of the surface electrostimulation controlled by muscle contraction on the bioelectric muscle activity and restoration of the hand function in cerebral stroke patients. NeuroRehabilitation. 2014;35(3):427–34.

231.
Kwon J-S, Park M-J, Yoon I-J, Park S-H. Effects of virtual reality on upper extremity function and activities of daily living performance in acute stroke: a double-blind randomized clinical trial. NeuroRehabilitation. 2012;31(4):379–85.

232.
Kwon TG, Kim Y-H, Chang WH, Bang OY, Shin Y-I. Effective method of combining rTMS and motor training in stroke patients. Restor Neurol Neurosci. 2014;32(2):223–32.

233.
Laible M, Grieshammer S, Seidel G, Rijntjes M, Weiller C, Hamzei F. Association of activity changes in the primary sensory cortex with successful motor rehabilitation of the hand following stroke. Neurorehabil Neural Repair. 2012 Sep;26(7):881–8.

234.
Lambercy O, Dovat L, Yun H, Wee SK, Kuah CWK, Chua KSG, et al. Effects of a robot-assisted training of grasp and pronation/supination in chronic stroke: a pilot study. J Neuroeng Rehabil. 2011;8:63.

235.
Langan J, van Donkelaar P. The influence of hand dominance on the response to a constraint-induced therapy program following stroke. Neurorehabil Neural Repair. 2008 Jun;22(3):298–304.

236.
Langhammer B, Lindmark B, Stanghelle JK. Stroke patients and long-term training: is it worthwhile? A randomized comparison of two different training strategies after rehabilitation. Clin Rehabil. 2007 Jun;21(6):495–510.

237.
Langhammer B, Lindmark B, Stanghelle JK. Physiotherapy and physical functioning post-stroke: exercise habits and functioning 4 years later? Long-term follow-up after a 1-year long-term intervention period: a randomized controlled trial. Brain Inj. 2014;28(11):1396–405.

238.
Langhammer B, Stanghelle JK, Lindmark B. An evaluation of two different exercise regimes during the first year following stroke: a randomised controlled trial. Physiother Theory Pract. 2009 Feb;25(2):55–68.

239.
Langhammer B, Stanghelle JK. Can physiotherapy after stroke based on the Bobath concept result in improved quality of movement compared to the motor relearning programme. Physiother Res Int. 2011 Jun;16(2):69–80.

240.
Lannin NA, Cusick A, McCluskey A, Herbert RD. Effects of splinting on wrist contracture after stroke: a randomized controlled trial. Stroke. 2007 Jan;38(1):111–6.

241.
Lazar RM, Berman MF, Festa JR, Geller AE, Matejovsky TG, Marshall RS. GABAergic but not anti-cholinergic agents re-induce clinical deficits after stroke. J Neurol Sci. 2010 May 15;292(1-2):72–6.

242.
Lee D, Lee M, Lee K, Song C. Asymmetric training using virtual reality reflection equipment and the enhancement of upper limb function in stroke patients: a randomized controlled trial. J Stroke Cerebrovasc Dis. 2014 Jul;23(6):1319–26.

243.
Lee MM, Cho H-Y, Song CH. The mirror therapy program enhances upper-limb motor recovery and motor function in acute stroke patients. Am J Phys Med Rehabil. 2012 Aug;91(8):689–96, quiz 697–700.

244.
Lefebvre S, Thonnard J-L, Laloux P, Peeters A, Jamart J, Vandermeeren Y. Single session of dual-tDCS transiently improves precision grip and dexterity of the paretic hand after stroke. Neurorehabil Neural Repair. 2014 Feb;28(2):100–10.

245.
Lemmens RJM, Timmermans AAA, Janssen-Potten YJM, Pulles SANTD, Geers RPJ, Bakx WGM, et al. Accelerometry measuring the outcome of robot-supported upper limb training in chronic stroke: a randomized controlled trial. PLoS ONE. 2014;9(5):e96414.

246.
Levy CE, Giuffrida C, Richards L, Wu S, Davis S, Nadeau SE. Botulinum toxin a, evidence-based exercise therapy, and constraint-induced movement therapy for upper-limb hemiparesis attributable to stroke: a preliminary study. Am J Phys Med Rehabil. 2007 Sep;86(9):696–706.

247.
Levy R, Ruland S, Weinand M, Lowry D, Dafer R, Bakay R. Cortical stimulation for the rehabilitation of patients with hemiparetic stroke: a multicenter feasibility study of safety and efficacy. J Neurosurg. 2008 Apr;108(4):707–14.

248.
Lewis GN, Woods C, Rosie JA, McPherson KM. Virtual reality games for rehabilitation of people with stroke: perspectives from the users. Disabil Rehabil Assist Technol. 2011;6(5):453–63.

249.
Liepert J, Binder C. Vibration-induced effects in stroke patients with spastic hemiparesis--a pilot study. Restor Neurol Neurosci. 2010;28(6):729–35.

250.
Liepert J, Greiner J, Dettmers C. Motor excitability changes during action observation in stroke patients. J Rehabil Med. 2014 May;46(5):400–5.

251.
Liepert J, Greiner J, Nedelko V, Dettmers C. Reduced upper limb sensation impairs mental chronometry for motor imagery after stroke: clinical and electrophysiological findings. Neurorehabil Neural Repair. 2012 Jun;26(5):470–8.

252.
Liepert J, Haevernick K, Weiller C, Barzel A. The surround inhibition determines therapy-induced cortical reorganization. Neuroimage. 2006 Sep;32(3):1216–20.

253.

Liepert J, Zittel S, Weiller C. Improvement of dexterity by single session low-frequency repetitive transcranial magnetic stimulation over the contralesional motor cortex in acute stroke: a double-blind placebo-controlled crossover trial. Restor Neurol Neurosci. 2007;25(5-6):461–5.

254.
Liepert J. Motor cortex excitability in stroke before and after constraint-induced movement therapy. Cogn Behav Neurol. 2006 Mar;19(1):41–7.

255.
Lim J-Y, Koh J-H, Paik N-J. Intramuscular botulinum toxin-A reduces hemiplegic shoulder pain: a randomized, double-blind, comparative study versus intraarticular triamcinolone acetonide. Stroke. 2008 Jan;39(1):126–31.

256.
Lin K-C, Wu C-Y, Wei T-H, Lee C-Y, Liu J-S. Effects of modified constraint-induced movement therapy on reach-to-grasp movements and functional performance after chronic stroke: a randomized controlled study. Clin Rehabil. 2007 Dec;21(12):1075–86.

257.
Lin K, Chang Y, Wu C, Chen Y. Effects of constraint-induced therapy versus bilateral arm training on motor performance, daily functions, and quality of life in stroke survivors. Neurorehabil Neural Repair. 2009 Jun;23(5):441–8.

258.
Lin K, Chuang L, Wu C, Hsieh Y, Chang W. Responsiveness and validity of three dexterous function measures in stroke rehabilitation. J Rehabil Res Dev. 2010;47(6):563–71.

259.
Lin K, Hsieh Y, Wu C, Chen C, Jang Y, Liu J. Minimal detectable change and clinically important difference of the Wolf Motor Function Test in stroke patients. Neurorehabil Neural Repair. 2009 Jun;23(5):429–34.

260.
Lin K, Wu C, Liu J, Chen Y, Hsu C. Constraint-induced therapy versus dose-matched control intervention to improve motor ability, basic/extended daily functions, and quality of life in stroke. Neurorehabil Neural Repair. 2009 Feb;23(2):160–5.

261.
Lindberg P, Schmitz C, Forssberg H, Engardt M, Borg J. Effects of passive-active movement training on upper limb motor function and cortical activation in chronic patients with stroke: a pilot study. J Rehabil Med. 2004 May;36(3):117–23.

262.
Lindberg PG, Roche N, Robertson J, Roby-Brami A, Bussel B, Maier MA. Affected and unaffected quantitative aspects of grip force control in hemiparetic patients after stroke. Brain Res. 2012 May 3;1452:96–107.

263.
Linder SM, Reiss A, Buchanan S, Sahu K, Rosenfeldt AB, Clark C, et al. Incorporating robotic-assisted telerehabilitation in a home program to improve arm function following stroke. J Neurol Phys Ther. 2013 Sep;37(3):125–32.

264.
Lodha N, Coombes SA, Cauraugh JH. Bimanual isometric force control: asymmetry and coordination evidence post stroke. Clin Neurophysiol. 2012 Apr;123(4):787–95.

265.
Loureiro RCV, Harwin WS, Lamperd R, Collin C. Evaluation of reach and grasp robot-assisted therapy suggests similar functional recovery patterns on proximal and distal arm segments in sub-acute hemiplegia. IEEE Trans Neural Syst Rehabil Eng. 2014 May;22(3):593–602.

266.
Lourenção MIP, Battistella LR, de Brito CMM, Tsukimoto GR, Miyazaki MH. Effect of biofeedback accompanying occupational therapy and functional electrical stimulation in hemiplegic patients. Int J Rehabil Res. 2008 Mar;31(1):33–41.

267.
Luft AR, McCombe-Waller S, Whitall J, Forrester LW, Macko R, Sorkin JD, et al. Repetitive bilateral arm training and motor cortex activation in chronic stroke: a randomized controlled trial. JAMA. 2004 Oct 20;292(15):1853–61.

268.
Lum PS, Taub E, Schwandt D, Postman M, Hardin P, Uswatte G. Automated Constraint-Induced Therapy Extension (AutoCITE) for movement deficits after stroke. J Rehabil Res Dev. 2004 May;41(3A):249–58.

269.
Lum PS, Uswatte G, Taub E, Hardin P, Mark VW. A telerehabilitation approach to delivery of constraint-induced movement therapy. J Rehabil Res Dev. 2006 Jun;43(3):391–400.

270.
Lundquist CB, Nielsen JF. Left/right judgement does not influence the effect of mirror therapy after stroke. Disabil Rehabil. 2014;36(17):1452–6.

271.
Makowski N, Knutson J, Chae J, Crago P. Interaction of poststroke voluntary effort and functional neuromuscular electrical stimulation. J Rehabil Res Dev. 2013;50(1):85–98.

272.
Malešević NM, Popović Maneski LZ, Ilić V, Jorgovanović N, Bijelić G, Keller T, et al. A multi-pad electrode based functional electrical stimulation system for restoration of grasp. J Neuroeng Rehabil. 2012;9:66.

273.
Malhotra S, Rosewilliam S, Hermens H, Roffe C, Jones P, Pandyan AD. A randomized controlled trial of surface neuromuscular electrical stimulation applied early after acute stroke: effects on wrist pain, spasticity and contractures. Clin Rehabil. 2013 Jul;27(7):579–90.

274.
Mali U, Goljar N, Munih M. Application of haptic interface for finger exercise. IEEE Trans Neural Syst Rehabil Eng. 2006 Sep;14(3):352–60.

275.

Manganotti P, Amelio E. Long-term effect of shock wave therapy on upper limb hypertonia in patients affected by stroke. Stroke. 2005 Sep;36(9):1967–71.

276.
Manganotti P, Acler M, Formaggio E, Avesani M, Milanese F, Baraldo A, et al. Changes in cerebral activity after decreased upper-limb hypertonus: an EMG-fMRI study. Magn Reson Imaging. 2010 Jun;28(5):646–52.

277.
Mangold S, Schuster C, Keller T, Zimmermann-Schlatter A, Ettlin T. Motor training of upper extremity with functional electrical stimulation in early stroke rehabilitation. Neurorehabil Neural Repair. 2009 Feb;23(2):184–90.

278.
Mansur CG, Fregni F, Boggio PS, Riberto M, Gallucci-Neto J, Santos CM, et al. A sham stimulation-controlled trial of rTMS of the unaffected hemisphere in stroke patients. Neurology. 2005 May 24;64(10):1802–4.

279.
Masiero S, Armani M, Ferlini G, Rosati G, Rossi A. Randomized trial of a robotic assistive device for the upper extremity during early inpatient stroke rehabilitation. Neurorehabil Neural Repair. 2014 May;28(4):377–86.

280.
Masiero S, Celia A, Rosati G, Armani M. Robotic-assisted rehabilitation of the upper limb after acute stroke. Arch Phys Med Rehabil. 2007 Feb;88(2):142–9.

281.
Mayer NH, Harvey RL. Use of a resting hand orthosis for the hemiparetic hand after stroke. PM R. 2014 Feb;6(2):188–95.

282.
McCabe J, Monkiewicz M, Holcomb J, Pundik S, Daly JJ. Comparison of robotics, functional electrical stimulation, and motor learning methods for treatment of persistent upper extremity dysfunction after stroke: a randomized controlled trial. Arch Phys Med Rehabil. 2015 Jun;96(6):981–90.

283.
McCombe Waller S, Whitall J. Hand dominance and side of stroke affect rehabilitation in chronic stroke. Clin Rehabil. 2005 Aug;19(5):544–51.

284.
McDonnell MN, Hillier SL, Esterman AJ. Standardizing the approach to evidence-based upper limb rehabilitation after stroke. Top Stroke Rehabil. 2013 Oct;20(5):432–40.

285.
McDonnell MN, Hillier SL, Miles TS, Thompson PD, Ridding MC. Influence of combined afferent stimulation and task-specific training following stroke: a pilot randomized controlled trial. Neurorehabil Neural Repair. 2007 Oct;21(5):435–43.

286.
Meadmore KL, Exell TA, Hallewell E, Hughes A-M, Freeman CT, Kutlu M, et al. The application of precisely controlled functional electrical stimulation to the shoulder, elbow and

wrist for upper limb stroke rehabilitation: a feasibility study. J Neuroeng Rehabil. 2014;11:105.

287.
Medée B, Bellaiche S, Revol P, Jacquin-Courtois S, Arsenault L, Guichard-Mayel A, et al. Constraint therapy versus intensive training: implications for motor control and brain plasticity after stroke. Neuropsychol Rehabil. 2010 Dec;20(6):854–68.

288.
Merians AS, Fluet GG, Qiu Q, Saleh S, Lafond I, Davidow A, et al. Robotically facilitated virtual rehabilitation of arm transport integrated with finger movement in persons with hemiparesis. J Neuroeng Rehabil. 2011;8:27.

289.
Merians AS, Poizner H, Boian R, Burdea G, Adamovich S. Sensorimotor training in a virtual reality environment: does it improve functional recovery poststroke? Neurorehabil Neural Repair. 2006 Jun;20(2):252–67.

290.
Michaelsen SM, Dannenbaum R, Levin MF. Task-specific training with trunk restraint on arm recovery in stroke: randomized control trial. Stroke. 2006 Jan;37(1):186–92.

291.
Michielsen ME, Selles RW, van der Geest JN, Eckhardt M, Yavuzer G, Stam HJ, et al. Motor recovery and cortical reorganization after mirror therapy in chronic stroke patients: a phase II randomized controlled trial. Neurorehabil Neural Repair. 2011 Apr;25(3):223–33.

292.
Mihara M, Hattori N, Hatakenaka M, Yagura H, Kawano T, Hino T, et al. Near-infrared spectroscopy-mediated neurofeedback enhances efficacy of motor imagery-based training in poststroke victims: a pilot study. Stroke. 2013 Apr;44(4):1091–8.

293.
Milot M-H, Spencer SJ, Chan V, Allington JP, Klein J, Chou C, et al. A crossover pilot study evaluating the functional outcomes of two different types of robotic movement training in chronic stroke survivors using the arm exoskeleton BONES. J Neuroeng Rehabil. 2013;10:112.

294.
Milot M-H, Spencer SJ, Chan V, Allington JP, Klein J, Chou C, et al. Corticospinal excitability as a predictor of functional gains at the affected upper limb following robotic training in chronic stroke survivors. Neurorehabil Neural Repair. 2014 Dec;28(9):819–27.

295.
Miscio G, Del Conte C, Pianca D, Colombo R, Panizza M, Schieppati M, et al. Botulinum toxin in post-stroke patients: stiffness modifications and clinical implications. J Neurol. 2004 Feb;251(2):189–96.

296.
Morris JH, van Wijck F, Joice S, Ogston SA, Cole I, MacWalter RS. A comparison of bilateral and unilateral upper-limb task training in early poststroke rehabilitation: a randomized controlled trial. Arch Phys Med Rehabil. 2008 Jul;89(7):1237–45.

297.
Morris JH, Van Wijck F. Responses of the less affected arm to bilateral upper limb task training in early rehabilitation after stroke: a randomized controlled trial. Arch Phys Med Rehabil. 2012 Jul;93(7):1129–37.

298.
Mouawad MR, Doust CG, Max MD, McNulty PA. Wii-based movement therapy to promote improved upper extremity function post-stroke: a pilot study. J Rehabil Med. 2011 May;43(6):527–33.

299.
Mukaino M, Ono T, Shindo K, Fujiwara T, Ota T, Kimura A, et al. Efficacy of brain-computer interface-driven neuromuscular electrical stimulation for chronic paresis after stroke. J Rehabil Med. 2014 Apr;46(4):378–82.

300.
Mukherjee M, Koutakis P, Siu K-C, Fayad PB, Stergiou N. Stroke survivors control the temporal structure of variability during reaching in dynamic environments. Ann Biomed Eng. 2013 Feb;41(2):366–76.

301.
Müller K, Bütefisch CM, Seitz RJ, Hömberg V. Mental practice improves hand function after hemiparetic stroke. Restor Neurol Neurosci. 2007;25(5-6):501–11.

302.
Murayama T, Numata K, Kawakami T, Tosaka T, Oga M, Oka N, et al. Changes in the brain activation balance in motor-related areas after constraint-induced movement therapy; a longitudinal fMRI study. Brain Inj. 2011;25(11):1047–57.

303.
Myint JMWW, Yuen GFC, Yu TKK, Kng CPL, Wong AMY, Chow KKC, et al. A study of constraint-induced movement therapy in subacute stroke patients in Hong Kong. Clin Rehabil. 2008 Feb;22(2):112–24.

304.
Nair DG, Fuchs A, Burkart S, Steinberg FL, Kelso J a. S. Assessing recovery in middle cerebral artery stroke using functional MRI. Brain Inj. 2005 Dec;19(13):1165–76.

305.
Nilsen DM, DiRusso T. Using mirror therapy in the home environment: a case report. Am J Occup Ther. 2014 Jun;68(3):e84–9.

306.
Noma T, Matsumoto S, Etoh S, Shimodozono M, Kawahira K. Anti-spastic effects of the direct application of vibratory stimuli to the spastic muscles of hemiplegic limbs in post-stroke patients. Brain Inj. 2009 Jul;23(7):623–31.

307.
Nott MT, Barden HLH, Baguley IJ. Goal attainment following upper-limb botulinum toxin-A injections: are we facilitating achievement of client-centred goals? J Rehabil Med. 2014 Oct;46(9):864–8.

308.

Nowak DA, Grefkes C, Dafotakis M, Eickhoff S, Küst J, Karbe H, et al. Effects of low-frequency repetitive transcranial magnetic stimulation of the contralesional primary motor cortex on movement kinematics and neural activity in subcortical stroke. Arch Neurol. 2008 Jun;65(6):741–7.

309.
Osu R, Otaka Y, Ushiba J, Sakata S, Yamaguchi T, Fujiwara T, et al. A pilot study of contralateral homonymous muscle activity simulated electrical stimulation in chronic hemiplegia. Brain Inj. 2012;26(9):1105–12.

310.
Page SJ, Harnish SM, Lamy M, Eliassen JC, Szaflarski JP. Affected arm use and cortical change in stroke patients exhibiting minimal hand movement. Neurorehabil Neural Repair. 2010 Feb;24(2):195–203.

311.
Page SJ, Hermann VH, Levine PG, Lewis E, Stein J, DePeel J. Portable neurorobotics for the severely affected arm in chronic stroke: a case study. J Neurol Phys Ther. 2011 Mar;35(1):41–6.

312.
Page SJ, Levine P, Hill V. Mental practice as a gateway to modified constraint-induced movement therapy: a promising combination to improve function. Am J Occup Ther. 2007 Jun;61(3):321–7.

313.
Page SJ, Levine P, Khoury JC. Modified constraint-induced therapy combined with mental practice: thinking through better motor outcomes. Stroke. 2009 Feb;40(2):551–4.

314.
Page SJ, Levine P, Leonard A. Mental practice in chronic stroke: results of a randomized, placebo-controlled trial. Stroke. 2007 Apr;38(4):1293–7.

315.
Page SJ, Levine P. Back from the brink: electromyography-triggered stimulation combined with modified constraint-induced movement therapy in chronic stroke. Arch Phys Med Rehabil. 2006 Jan;87(1):27–31.

316.
Page SJ, Levine P. Modified constraint-induced therapy in patients with chronic stroke exhibiting minimal movement ability in the affected arm. Phys Ther. 2007 Jul;87(7):872–8.

317.
Page SJ, Murray C, Hermann V, Levine P. Retention of motor changes in chronic stroke survivors who were administered mental practice. Arch Phys Med Rehabil. 2011 Nov;92(11):1741–5.

318.
Pandian S, Arya KN, Davidson EWR. Comparison of Brunnstrom movement therapy and Motor Relearning Program in rehabilitation of post-stroke hemiparetic hand: a randomized trial. J Bodyw Mov Ther. 2012 Jul;16(3):330–7.

319.

Pang MY, Harris JE, Eng JJ. A community-based upper-extremity group exercise program improves motor function and performance of functional activities in chronic stroke: a randomized controlled trial. Arch Phys Med Rehabil. 2006 Jan;87(1):1–9.

320.
Park S-W, Butler AJ, Cavalheiro V, Alberts JL, Wolf SL. Changes in serial optical topography and TMS during task performance after constraint-induced movement therapy in stroke: a case study. Neurorehabil Neural Repair. 2004 Jun;18(2):95–105.

321.
Patel AT. Successful treatment of long-term, poststroke, upper-limb spasticity with onabotulinumtoxinA. Phys Ther. 2011 Nov;91(11):1636–41.

322.
Patten C, Dozono J, Schmidt S, Jue M, Lum P. Combined functional task practice and dynamic high intensity resistance training promotes recovery of upper-extremity motor function in post-stroke hemiparesis: a case study. J Neurol Phys Ther. 2006 Sep;30(3):99–115.

323.
Pellegrino G, Tomasevic L, Tombini M, Assenza G, Bravi M, Sterzi S, et al. Inter-hemispheric coupling changes associate with motor improvements after robotic stroke rehabilitation. Restor Neurol Neurosci. 2012;30(6):497–510.

324.
Picelli A, Lobba D, Midiri A, Prandi P, Melotti C, Baldessarelli S, et al. Botulinum toxin injection into the forearm muscles for wrist and fingers spastic overactivity in adults with chronic stroke: a randomized controlled trial comparing three injection techniques. Clin Rehabil. 2014 Mar;28(3):232–42.

325.
Pichiorri F, Morone G, Petti M, Toppi J, Pisotta I, Molinari M, et al. Brain-computer interface boosts motor imagery practice during stroke recovery. Ann Neurol. 2015 May;77(5):851–65.

326.
Pinter D, Pegritz S, Pargfrieder C, Reiter G, Wurm W, Gattringer T, et al. Exploratory study on the effects of a robotic hand rehabilitation device on changes in grip strength and brain activity after stroke. Top Stroke Rehabil. 2013 Aug;20(4):308–16.

327.
Pizzi A, Carlucci G, Falsini C, Verdesca S, Grippo A. Application of a volar static splint in poststroke spasticity of the upper limb. Arch Phys Med Rehabil. 2005 Sep;86(9):1855–9.

328.
Ploughman M, Corbett D. Can forced-use therapy be clinically applied after stroke? An exploratory randomized controlled trial. Arch Phys Med Rehabil. 2004 Sep;85(9):1417–23.

329.
Popovic DB, Popovic MB, Sinkjaer T, Stefanovic A, Schwirtlich L. Therapy of paretic arm in hemiplegic subjects augmented with a neural prosthesis: a cross-over study. Can J Physiol Pharmacol. 2004 Sep;82(8-9):749–56.

330.

Prange GB, Kottink AIR, Buurke JH, Eckhardt MMEM, van Keulen-Rouweler BJ, Ribbers GM, et al. The effect of arm support combined with rehabilitation games on upper-extremity function in subacute stroke: a randomized controlled trial. Neurorehabil Neural Repair. 2015 Feb;29(2):174–82.

331.
Prange GB, Krabben T, Renzenbrink GJ, Ijzerman MJ, Hermens HJ, Jannink MJA. Changes in muscle activation after reach training with gravity compensation in chronic stroke patients. Int J Rehabil Res. 2012 Sep;35(3):234–42.

332.
Proffitt RM, Alankus G, Kelleher CL, Engsberg JR. Use of computer games as an intervention for stroke. Top Stroke Rehabil. 2011 Aug;18(4):417–27.

333.
Puthenveettil S, Fluet G, Qiu Q, Adamovich S. Classification of hand preshaping in persons with stroke using Linear Discriminant Analysis. Conf Proc IEEE Eng Med Biol Soc. 2012;2012:4563–6.

334.
Qiu Q, Fluet GG, Lafond I, Merians AS, Adamovich SV. Coordination changes demonstrated by subjects with hemiparesis performing hand-arm training using the NJIT-RAVR robotically assisted virtual rehabilitation system. Conf Proc IEEE Eng Med Biol Soc. 2009;2009:1143–6.

335.
Ramos-Murguialday A, Broetz D, Rea M, Läer L, Yilmaz O, Brasil FL, et al. Brain-machine interface in chronic stroke rehabilitation: a controlled study. Ann Neurol. 2013 Jul;74(1):100–8.

336.
Ratmansky M, Defrin R, Soroker N. A randomized controlled study of segmental neuromyotherapy for post-stroke hemiplegic shoulder pain. J Rehabil Med. 2012 Oct;44(10):830–6.

337.
Rayegani SM, Raeissadat SA, Sedighipour L, Rezazadeh IM, Bahrami MH, Eliaspour D, et al. Effect of neurofeedback and electromyographic-biofeedback therapy on improving hand function in stroke patients. Top Stroke Rehabil. 2014 Apr;21(2):137–51.

338.
Renner CIE, Schubert M, Jahn M, Hummelsheim H. Intracortical excitability after repetitive hand movements is differentially affected in cortical versus subcortical strokes. J Clin Neurophysiol. 2009 Oct;26(5):348–57.

339.
Repšaitė V, Vainoras A, Berškienė K, Baltaduonienė D, Daunoravičienė A, Sendžikaitė E. The effect of differential training-based occupational therapy on hand and arm function in patients after stroke: Results of the pilot study. Neurol Neurochir Pol. 2015;49(3):150–5.

340.
Richards L, Gonzalez Rothi LJ, Davis S, Wu SS, Nadeau SE. Limited dose response to constraint-induced movement therapy in patients with chronic stroke. Clin Rehabil. 2006 Dec;20(12):1066–74.

341.
Rickards T, Sterling C, Taub E, Perkins-Hu C, Gauthier L, Graham M, et al. Diffusion tensor imaging study of the response to constraint-induced movement therapy of children with hemiparetic cerebral palsy and adults with chronic stroke. Arch Phys Med Rehabil. 2014 Mar;95(3):506–14.e1.

342.
Rickards T, Taub E, Sterling C, Graham MJ, Barghi A, Uswatte G, et al. Brain parenchymal fraction predicts motor improvement following intensive task-oriented motor rehabilitation for chronic stroke. Restor Neurol Neurosci. 2012;30(5):355–61.

343.
Ro T, Noser E, Boake C, Johnson R, Gaber M, Speroni A, et al. Functional reorganization and recovery after constraint-induced movement therapy in subacute stroke: case reports. Neurocase. 2006 Feb;12(1):50–60.

344.
Rosales RL, Kong KH, Goh KJ, Kumthornthip W, Mok VCT, Delgado-De Los Santos MM, et al. Botulinum toxin injection for hypertonicity of the upper extremity within 12 weeks after stroke: a randomized controlled trial. Neurorehabil Neural Repair. 2012 Sep;26(7):812–21.

345.
Rosenstein L, Ridgel AL, Thota A, Samame B, Alberts JL. Effects of combined robotic therapy and repetitive-task practice on upper-extremity function in a patient with chronic stroke. Am J Occup Ther. 2008 Feb;62(1):28–35.

346.
Rosewilliam S, Malhotra S, Roffe C, Jones P, Pandyan AD. Can surface neuromuscular electrical stimulation of the wrist and hand combined with routine therapy facilitate recovery of arm function in patients with stroke? Arch Phys Med Rehabil. 2012 Oct;93(10):1715–21.e1.

347.
Ross LF, Harvey LA, Lannin NA. Do people with acquired brain impairment benefit from additional therapy specifically directed at the hand? A randomized controlled trial. Clin Rehabil. 2009 Jun;23(6):492–503.

348.
Rowe VT, Blanton S, Wolf SL. Long-term follow-up after constraint-induced therapy: a case report of a chronic stroke survivor. Am J Occup Ther. 2009 Jun;63(3):317–22.

349.
Runnarong N, Tretriluxana J, Hiengkaew V, Vachalathiti R. Reach-to-grasp co-ordination in the paretic limbs of individuals with stroke: insight from a barrier paradigm. J Med Assoc Thai. 2014 Jul;97 Suppl 7:S84–8.

350.
Sale P, Ceravolo MG, Franceschini M. Action observation therapy in the subacute phase promotes dexterity recovery in right-hemisphere stroke patients. Biomed Res Int. 2014;2014:457538.

351.

Sale P, Mazzoleni S, Lombardi V, Galafate D, Massimiani MP, Posteraro F, et al. Recovery of hand function with robot-assisted therapy in acute stroke patients: a randomized-controlled trial. Int J Rehabil Res. 2014 Sep;37(3):236–42.

352.
Saleh S, Bagce H, Qiu Q, Fluet G, Merians A, Adamovich S, et al. Mechanisms of neural reorganization in chronic stroke subjects after virtual reality training. Conf Proc IEEE Eng Med Biol Soc. 2011;2011:8118–21.

353.
Saleh S, Adamovich SV, Tunik E. Resting state functional connectivity and task-related effective connectivity changes after upper extremity rehabilitation: a pilot study. Conf Proc IEEE Eng Med Biol Soc. 2012;2012:4559–62.

354.
Samuelkamaleshkumar S, Reethajanetsureka S, Pauljebaraj P, Benshamir B, Padankatti SM, David JA. Mirror therapy enhances motor performance in the paretic upper limb after stroke: a pilot randomized controlled trial. Arch Phys Med Rehabil. 2014 Nov;95(11):2000–5.

355.
Sanchez RJ, Liu J, Rao S, Shah P, Smith R, Rahman T, et al. Automating arm movement training following severe stroke: functional exercises with quantitative feedback in a gravity-reduced environment. IEEE Trans Neural Syst Rehabil Eng. 2006 Sep;14(3):378–89.

356.
Santamato A, Micello MF, Panza F, Fortunato F, Baricich A, Cisari C, et al. Can botulinum toxin type A injection technique influence the clinical outcome of patients with post-stroke upper limb spasticity? A randomized controlled trial comparing manual needle placement and ultrasound-guided injection techniques. J Neurol Sci. 2014 Dec 15;347(1-2):39–43.

357.
Santamato A, Panza F, Filoni S, Ranieri M, Solfrizzi V, Frisardi V, et al. Effect of botulinum toxin type A, motor imagery and motor observation on motor function of hemiparetic upper limb after stroke. Brain Inj. 2010;24(9):1108–12.

358.
Sasaki N, Kakuda W, Abo M. Bilateral high- and low-frequency rTMS in acute stroke patients with hemiparesis: a comparative study with unilateral high-frequency rTMS. Brain Inj. 2014;28(13-14):1682–6.

359.
Sawaki L, Butler AJ, Leng X, Wassenaar PA, Mohammad YM, Blanton S, et al. Constraint-induced movement therapy results in increased motor map area in subjects 3 to 9 months after stroke. Neurorehabil Neural Repair. 2008 Oct;22(5):505–13.

360.
Sawaki L, Wu CW-H, Kaelin-Lang A, Cohen LG. Effects of somatosensory stimulation on use-dependent plasticity in chronic stroke. Stroke. 2006 Jan;37(1):246–7.

361.
Schuster C, Maunz G, Lutz K, Kischka U, Sturzenegger R, Ettlin T. Dexamphetamine improves upper extremity outcome during rehabilitation after stroke: a pilot randomized controlled trial. Neurorehabil Neural Repair. 2011 Oct;25(8):749–55.

362.
Senesac CR, Davis S, Richards L. Generalization of a modified form of repetitive rhythmic bilateral training in stroke. Hum Mov Sci. 2010 Feb;29(1):137–48.

363.
Seniów J, Bilik M, Leśniak M, Waldowski K, Iwański S, Członkowska A. Transcranial magnetic stimulation combined with physiotherapy in rehabilitation of poststroke hemiparesis: a randomized, double-blind, placebo-controlled study. Neurorehabil Neural Repair. 2012 Dec;26(9):1072–9.

364.
Senkárová Z, Hlustík P, Otruba P, Herzig R, Kanovský P. Modulation of cortical activity in patients suffering from upper arm spasticity following stroke and treated with botulinum toxin A: an fMRI study. J Neuroimaging. 2010 Jan;20(1):9–15.

365.
Sens E, Knorr C, Preul C, Meissner W, Witte OW, Miltner WHR, et al. Differences in somatosensory and motor improvement during temporary functional deafferentation in stroke patients and healthy subjects. Behav Brain Res. 2013 Sep 1;252:110–6.

366.
Sens E, Teschner U, Meissner W, Preul C, Huonker R, Witte OW, et al. Effects of temporary functional deafferentation on the brain, sensation, and behavior of stroke patients. J Neurosci. 2012 Aug 22;32(34):11773–9.

367.
Seo NJ, Fischer HW, Bogey RA, Rymer WZ, Kamper DG. Use of visual force feedback to improve digit force direction during pinch grip in persons with stroke: a pilot study. Arch Phys Med Rehabil. 2011 Jan;92(1):24–30.

368.
Seo NJ, Fischer HW, Bogey RA, Rymer WZ, Kamper DG. Effect of a serotonin antagonist on delay in grip muscle relaxation for persons with chronic hemiparetic stroke. Clin Neurophysiol. 2011 Apr;122(4):796–802.

369.
Seo NJ, Kamper DG. Effect of grip location, arm support, and muscle stretch on sustained finger flexor activity following stroke. Conf Proc IEEE Eng Med Biol Soc. 2008;2008:4170–3.

370.
Seo NJ, Rymer WZ, Kamper DG. Delays in grip initiation and termination in persons with stroke: effects of arm support and active muscle stretch exercise. J Neurophysiol. 2009 Jun;101(6):3108–15.

371.
Shaw L, Rodgers H, Price C, van Wijck F, Shackley P, Steen N, et al. BoTULS: a multicentre randomised controlled trial to evaluate the clinical effectiveness and cost-effectiveness of treating upper limb spasticity due to stroke with botulinum toxin type A. Health Technol Assess. 2010 May;14(26):1–113, iii – iv.

372.

Shaw LC, Price CIM, van Wijck FMJ, Shackley P, Steen N, Barnes MP, et al. Botulinum Toxin for the Upper Limb after Stroke (BoTULS) Trial: effect on impairment, activity limitation, and pain. Stroke. 2011 May;42(5):1371–9.

373.
Shin HK, Cho SH, Jeon H, Lee Y-H, Song JC, Jang SH, et al. Cortical effect and functional recovery by the electromyography-triggered neuromuscular stimulation in chronic stroke patients. Neurosci Lett. 2008 Sep 19;442(3):174–9.

374.
Shin J-H, Ryu H, Jang SH. A task-specific interactive game-based virtual reality rehabilitation system for patients with stroke: a usability test and two clinical experiments. J Neuroeng Rehabil. 2014;11:32.

375.
Shindo K, Fujiwara T, Hara J, Oba H, Hotta F, Tsuji T, et al. Effectiveness of hybrid assistive neuromuscular dynamic stimulation therapy in patients with subacute stroke: a randomized controlled pilot trial. Neurorehabil Neural Repair. 2011 Dec;25(9):830–7.

376.
Shindo K, Kawashima K, Ushiba J, Ota N, Ito M, Ota T, et al. Effects of neurofeedback training with an electroencephalogram-based brain-computer interface for hand paralysis in patients with chronic stroke: a preliminary case series study. J Rehabil Med. 2011 Oct;43(10):951–7.

377.
Shiri S, Feintuch U, Lorber-Haddad A, Moreh E, Twito D, Tuchner-Arieli M, et al. Novel virtual reality system integrating online self-face viewing and mirror visual feedback for stroke rehabilitation: rationale and feasibility. Top Stroke Rehabil. 2012 Aug;19(4):277–86.

378.
Siebers A, Oberg U, Skargren E. The effect of modified constraint-induced movement therapy on spasticity and motor function of the affected arm in patients with chronic stroke. Physiother Can. 2010;62(4):388–96.

379.
Simpson DM, Gracies JM, Yablon SA, Barbano R, Brashear A, BoNT/TZD Study Team. Botulinum neurotoxin versus tizanidine in upper limb spasticity: a placebo-controlled study. J Neurol Neurosurg Psychiatr. 2009 Apr;80(4):380–5.

380.
Sin H, Lee G. Additional virtual reality training using Xbox Kinect in stroke survivors with hemiplegia. Am J Phys Med Rehabil. 2013 Oct;92(10):871–80.

381.
Slawek J, Bogucki A, Reclawowicz D. Botulinum toxin type A for upper limb spasticity following stroke: an open-label study with individualised, flexible injection regimens. Neurol Sci. 2005 Apr;26(1):32–9.

382.
Slijper A, Svensson KE, Backlund P, Engström H, Sunnerhagen KS. Computer game-based upper extremity training in the home environment in stroke persons: a single subject design. J Neuroeng Rehabil. 2014;11:35.

383.
Smania N, Gandolfi M, Paolucci S, Iosa M, Ianes P, Recchia S, et al. Reduced-intensity modified constraint-induced movement therapy versus conventional therapy for upper extremity rehabilitation after stroke: a multicenter trial. Neurorehabil Neural Repair. 2012 Dec;26(9):1035–45.

384.
Smedes F, van der Salm A, Koel G, Oosterveld F. Manual mobilization of the wrist: a pilot study in rehabilitation of patients with a chronic hemiplegic hand post-stroke. J Hand Ther. 2014 Sep;27(3):209–15; quiz 216.

385.
Smith PS, Dinse HR, Kalisch T, Johnson M, Walker-Batson D. Effects of repetitive electrical stimulation to treat sensory loss in persons poststroke. Arch Phys Med Rehabil. 2009 Dec;90(12):2108–11.

386.
Squeri V, Masia L, Giannoni P, Sandini G, Morasso P. Wrist rehabilitation in chronic stroke patients by means of adaptive, progressive robot-aided therapy. IEEE Trans Neural Syst Rehabil Eng. 2014 Mar;22(2):312–25.

387.
Stagg CJ, Bachtiar V, O'Shea J, Allman C, Bosnell RA, Kischka U, et al. Cortical activation changes underlying stimulation-induced behavioural gains in chronic stroke. Brain. 2012 Jan;135(Pt 1):276–84.

388.
Stein J, Bishop L, Gillen G, Helbok R. Robot-assisted exercise for hand weakness after stroke: a pilot study. Am J Phys Med Rehabil. 2011 Nov;90(11):887–94.

389.
Stevens JA, Stoykov MEP. Simulation of bilateral movement training through mirror reflection: a case report demonstrating an occupational therapy technique for hemiparesis. Top Stroke Rehabil. 2004;11(1):59–66.

390.
Stevenson T, Thalman L. A modified constraint-induced movement therapy regimen for individuals with upper extremity hemiplegia. Can J Occup Ther. 2007 Apr;74(2):115–24.

391.
Stewart JC, Yeh S-C, Jung Y, Yoon H, Whitford M, Chen S-Y, et al. Intervention to enhance skilled arm and hand movements after stroke: A feasibility study using a new virtual reality system. J Neuroeng Rehabil. 2007;4:21.

392.
Stinear CM, Petoe MA, Anwar S, Barber PA, Byblow WD. Bilateral priming accelerates recovery of upper limb function after stroke: a randomized controlled trial. Stroke. 2014 Jan;45(1):205–10.

393.
Stowe AM, Hughes-Zahner L, Barnes VK, Herbelin LL, Schindler-Ivens SM, Quaney BM. A pilot study to measure upper extremity H-reflexes following neuromuscular electrical stimulation therapy after stroke. Neurosci Lett. 2013 Feb 22;535:1–6.

394.
Stoykov ME, Lewis GN, Corcos DM. Comparison of bilateral and unilateral training for upper extremity hemiparesis in stroke. Neurorehabil Neural Repair. 2009 Nov;23(9):945–53.

395.
Sullivan JE, Hurley D, Hedman LD. Afferent stimulation provided by glove electrode during task-specific arm exercise following stroke. Clin Rehabil. 2012 Nov;26(11):1010–20.

396.
Sun S-F, Hsu C-W, Hwang C-W, Hsu P-T, Wang J-L, Yang C-L. Application of combined botulinum toxin type A and modified constraint-induced movement therapy for an individual with chronic upper-extremity spasticity after stroke. Phys Ther. 2006 Oct;86(10):1387–97.

397.
Sun S-F, Hsu C-W, Sun H-P, Hwang C-W, Yang C-L, Wang J-L. Combined botulinum toxin type A with modified constraint-induced movement therapy for chronic stroke patients with upper extremity spasticity: a randomized controlled study. Neurorehabil Neural Repair. 2010 Jan;24(1):34–41.

398.
Szaflarski JP, Page SJ, Kissela BM, Lee J-H, Levine P, Strakowski SM. Cortical reorganization following modified constraint-induced movement therapy: a study of 4 patients with chronic stroke. Arch Phys Med Rehabil. 2006 Aug;87(8):1052–8.

399.
Taheri H, Rowe JB, Gardner D, Chan V, Reinkensmeyer DJ, Wolbrecht ET. Robot-assisted Guitar Hero for finger rehabilitation after stroke. Conf Proc IEEE Eng Med Biol Soc. 2012;2012:3911–7.

400.
Takahashi CD, Der-Yeghiaian L, Le V, Motiwala RR, Cramer SC. Robot-based hand motor therapy after stroke. Brain. 2008 Feb;131(Pt 2):425–37.

401.
Takebayashi T, Amano S, Hanada K, Umeji A, Takahashi K, Koyama T, et al. Therapeutic synergism in the treatment of post-stroke arm paresis utilizing botulinum toxin, robotic therapy, and constraint-induced movement therapy. PM R. 2014 Nov;6(11):1054–8.

402.
Takebayashi T, Koyama T, Amano S, Hanada K, Tabusadani M, Hosomi M, et al. A 6-month follow-up after constraint-induced movement therapy with and without transfer package for patients with hemiparesis after stroke: a pilot quasi-randomized controlled trial. Clin Rehabil. 2013 May;27(5):418–26.

403.
Takekawa T, Kakuda W, Taguchi K, Ishikawa A, Sase Y, Abo M. Botulinum toxin type A injection, followed by home-based functional training for upper limb hemiparesis after stroke. Int J Rehabil Res. 2012 Jun;35(2):146–52.

404.
Takeuchi N, Chuma T, Matsuo Y, Watanabe I, Ikoma K. Repetitive transcranial magnetic stimulation of contralesional primary motor cortex improves hand function after stroke. Stroke. 2005 Dec;36(12):2681–6.

405.
Takeuchi N, Tada T, Matsuo Y, Ikoma K. Low-frequency repetitive TMS plus anodal transcranial DCS prevents transient decline in bimanual movement induced by contralesional inhibitory rTMS after stroke. Neurorehabil Neural Repair. 2012 Oct;26(8):988–98.

406.
Takeuchi N, Tada T, Toshima M, Chuma T, Matsuo Y, Ikoma K. Inhibition of the unaffected motor cortex by 1 Hz repetitive transcranical magnetic stimulation enhances motor performance and training effect of the paretic hand in patients with chronic stroke. J Rehabil Med. 2008 Apr;40(4):298–303.

407.
Takeuchi N, Tada T, Toshima M, Matsuo Y, Ikoma K. Repetitive transcranial magnetic stimulation over bilateral hemispheres enhances motor function and training effect of paretic hand in patients after stroke. J Rehabil Med. 2009 Nov;41(13):1049–54.

408.
Takeuchi N, Toshima M, Chuma T, Matsuo Y, Ikoma K. Repetitive transcranial magnetic stimulation of the unaffected hemisphere in a patient who was forced to use the affected hand. Am J Phys Med Rehabil. 2008 Jan;87(1):74–7.

409.
Talelli P, Greenwood RJ, Rothwell JC. Exploring Theta Burst Stimulation as an intervention to improve motor recovery in chronic stroke. Clin Neurophysiol. 2007 Feb;118(2):333–42.

410.
Tan C, Tretriluxana J, Pitsch E, Runnarong N, Winstein CJ. Anticipatory planning of functional reach-to-grasp: a pilot study. Neurorehabil Neural Repair. 2012 Oct;26(8):957–67.

411.
Tardy J, Pariente J, Leger A, Dechaumont-Palacin S, Gerdelat A, Guiraud V, et al. Methylphenidate modulates cerebral post-stroke reorganization. Neuroimage. 2006 Nov 15;33(3):913–22.

412.
Tarkka IM, Pitkänen K, Popovic DB, Vanninen R, Könönen M. Functional electrical therapy for hemiparesis alleviates disability and enhances neuroplasticity. Tohoku J Exp Med. 2011;225(1):71–6.

413.
Tarkka IM, Pitkänen K, Sivenius J. Paretic hand rehabilitation with constraint-induced movement therapy after stroke. Am J Phys Med Rehabil. 2005 Jul;84(7):501–5.

414.
Taub E, Lum PS, Hardin P, Mark VW, Uswatte G. AutoCITE: automated delivery of CI therapy with reduced effort by therapists. Stroke. 2005 Jun;36(6):1301–4.

415.
Taub E, Uswatte G, Bowman MH, Mark VW, Delgado A, Bryson C, et al. Constraint-induced movement therapy combined with conventional neurorehabilitation techniques in chronic stroke patients with plegic hands: a case series. Arch Phys Med Rehabil. 2013 Jan;94(1):86–94.

416.
Theilig S, Podubecka J, Bösl K, Wiederer R, Nowak DA. Functional neuromuscular stimulation to improve severe hand dysfunction after stroke: does inhibitory rTMS enhance therapeutic efficiency? Exp Neurol. 2011 Jul;230(1):149–55.

417.
Thielman GT, Dean CM, Gentile AM. Rehabilitation of reaching after stroke: task-related training versus progressive resistive exercise. Arch Phys Med Rehabil. 2004 Oct;85(10):1613–8.

418.
Thielman G. Insights into upper limb kinematics and trunk control one year after task-related training in chronic post-stroke individuals. J Hand Ther. 2013 Jun;26(2):156–60; quiz 161.

419.
Thrasher TA, Zivanovic V, McIlroy W, Popovic MR. Rehabilitation of reaching and grasping function in severe hemiplegic patients using functional electrical stimulation therapy. Neurorehabil Neural Repair. 2008 Dec;22(6):706–14.

420.
Timmermans AAA, Lemmens RJM, Monfrance M, Geers RPJ, Bakx W, Smeets RJEM, et al. Effects of task-oriented robot training on arm function, activity, and quality of life in chronic stroke patients: a randomized controlled trial. J Neuroeng Rehabil. 2014;11:45.

421.
Timmermans AAA, Seelen HAM, Geers RPJ, Saini PK, Winter S, Vrugt J te, et al. Sensor-based arm skill training in chronic stroke patients: results on treatment outcome, patient motivation, and system usability. IEEE Trans Neural Syst Rehabil Eng. 2010 Jun;18(3):284–92.

422.
Timmermans AAA, Verbunt JA, van Woerden R, Moennekens M, Pernot DH, Seelen HAM. Effect of mental practice on the improvement of function and daily activity performance of the upper extremity in patients with subacute stroke: a randomized clinical trial. J Am Med Dir Assoc. 2013 Mar;14(3):204–12.

423.
Toole JF, Flowers DL, Burdette JH, Absher JR. A pianist's recovery from stroke. Arch Neurol. 2007 Aug;64(8):1184–8.

424.
Treger I, Aidinof L, Lehrer H, Kalichman L. Modified constraint-induced movement therapy improved upper limb function in subacute poststroke patients: a small-scale clinical trial. Top Stroke Rehabil. 2012 Aug;19(4):287–93.

425.
Tretriluxana J, Kantak S, Tretriluxana S, Wu AD, Fisher BE. Low frequency repetitive transcranial magnetic stimulation to the non-lesioned hemisphere improves paretic arm reach-to-grasp performance after chronic stroke. Disabil Rehabil Assist Technol. 2013 Mar;8(2):121–4.

426.

Tretriluxana J, Runnarong N, Tretriluxana S, Prayoonwiwat N, Vachalathiti R, Winstein C. Feasibility investigation of the Accelerated Skill Acquisition Program (ASAP): insights into reach-to-grasp coordination of individuals with postacute stroke. Top Stroke Rehabil. 2013 Apr;20(2):151–60.

427. Triandafilou KM, Kamper DG. Carryover effects of cyclical stretching of the digits on hand function in stroke survivors. Arch Phys Med Rehabil. 2014 Aug;95(8):1571–6.

428. Triandafilou KM, Ochoa J, Kang X, Fischer HC, Stoykov ME, Kamper DG. Transient impact of prolonged versus repetitive stretch on hand motor control in chronic stroke. Top Stroke Rehabil. 2011 Aug;18(4):316–24.

429. Troncati F, Paci M, Myftari T, Lombardi B. Extracorporeal Shock Wave Therapy reduces upper limb spasticity and improves motricity in patients with chronic hemiplegia: a case series. NeuroRehabilitation. 2013;33(3):399–405.

430. Tsoupikova D, Stoykov N, Kamper D, Vick R. Virtual Reality environment assisting post stroke hand rehabilitation: case report. Stud Health Technol Inform. 2013;184:458–64.

431. Tsoupikova D, Stoykov NS, Corrigan M, Thielbar K, Vick R, Li Y, et al. Virtual immersion for post-stroke hand rehabilitation therapy. Ann Biomed Eng. 2015 Feb;43(2):467–77.

432. Tunik E, Adamovich SV. Remapping in the ipsilesional motor cortex after VR-based training: a pilot fMRI study. Conf Proc IEEE Eng Med Biol Soc. 2009;2009:1139–42.

433. Tunik E, Saleh S, Adamovich SV. Visuomotor discordance during visually-guided hand movement in virtual reality modulates sensorimotor cortical activity in healthy and hemiparetic subjects. IEEE Trans Neural Syst Rehabil Eng. 2013 Mar;21(2):198–207.

434. Turton AJ, Britton E. A pilot randomized controlled trial of a daily muscle stretch regime to prevent contractures in the arm after stroke. Clin Rehabil. 2005 Sep;19(6):600–12.

435. Turton AJ, Butler SR. A multiple case design experiment to investigate the performance and neural effects of a programme for training hand function after stroke. Clin Rehabil. 2004 Nov;18(7):754–63.

436. Urbin MA, Waddell KJ, Lang CE. Acceleration metrics are responsive to change in upper extremity function of stroke survivors. Arch Phys Med Rehabil. 2015 May;96(5):854–61.

437. Uswatte G, Taub E, Morris D, Barman J, Crago J. Contribution of the shaping and restraint components of Constraint-Induced Movement therapy to treatment outcome. NeuroRehabilitation. 2006;21(2):147–56.

438.
van Delden ALEQ, Peper CLE, Nienhuys KN, Zijp NI, Beek PJ, Kwakkel G. Unilateral versus bilateral upper limb training after stroke: the Upper Limb Training After Stroke clinical trial. Stroke. 2013 Sep;44(9):2613–6.

439.
van den Hoogen W, Feys P, Lamers I, Coninx K, Notelaers S, Kerkhofs L, et al. Visualizing the third dimension in virtual training environments for neurologically impaired persons: beneficial or disruptive? J Neuroeng Rehabil. 2012;9:73.

440.
van der Kooij H, Prange GB, Krabben T, Renzenbrink GJ, de Boer J, Hermens HJ, et al. Preliminary results of training with gravity compensation of the arm in chronic stroke survivors. Conf Proc IEEE Eng Med Biol Soc. 2009;2009:2426–9.

441.
van Kordelaar J, van Wegen EEH, Nijland RHM, de Groot JH, Meskers CGM, Harlaar J, et al. Assessing longitudinal change in coordination of the paretic upper limb using on-site 3-dimensional kinematic measurements. Phys Ther. 2012 Jan;92(1):142–51.

442.
van Vliet PM, Lincoln NB, Foxall A. Comparison of Bobath based and movement science based treatment for stroke: a randomised controlled trial. J Neurol Neurosurg Psychiatr. 2005 Apr;76(4):503–8.

443.
Várkuti B, Guan C, Pan Y, Phua KS, Ang KK, Kuah CWK, et al. Resting state changes in functional connectivity correlate with movement recovery for BCI and robot-assisted upper-extremity training after stroke. Neurorehabil Neural Repair. 2013 Jan;27(1):53–62.

444.
Veverka T, Hluštík P, Hok P, Otruba P, Tüdös Z, Zapletalová J, et al. Cortical activity modulation by botulinum toxin type A in patients with post-stroke arm spasticity: real and imagined hand movement. J Neurol Sci. 2014 Nov 15;346(1-2):276–83.

445.
Vongvaivanichakul P, Tretriluxana J, Bovonsunthonchai S, Pakaprot N, Laksanakorn W. Reach-to-grasp training in individuals with chronic stroke augmented by low-frequency repetitive transcranial magnetic stimulation. J Med Assoc Thai. 2014 Jul;97 Suppl 7:S45–9.

446.
von Lewinski F, Hofer S, Kaus J, Merboldt K-D, Rothkegel H, Schweizer R, et al. Efficacy of EMG-triggered electrical arm stimulation in chronic hemiparetic stroke patients. Restor Neurol Neurosci. 2009;27(3):189–97.

447.
Wang B-H, Lin C-L, Li T-M, Lin S-D, Lin J-G, Chou L-W. Selection of acupoints for managing upper-extremity spasticity in chronic stroke patients. Clin Interv Aging. 2014;9:147–56.

448.

Wang C-P, Tsai P-Y, Yang TF, Yang K-Y, Wang C-C. Differential effect of conditioning sequences in coupling inhibitory/facilitatory repetitive transcranial magnetic stimulation for poststroke motor recovery. CNS Neurosci Ther. 2014 Apr;20(4):355–63.

449.
Wang LE, Fink GR, Diekhoff S, Rehme AK, Eickhoff SB, Grefkes C. Noradrenergic enhancement improves motor network connectivity in stroke patients. Ann Neurol. 2011 Feb;69(2):375–88.

450.
Wang Q, Zhao J-L, Zhu Q-X, Li J, Meng P-P. Comparison of conventional therapy, intensive therapy and modified constraint-induced movement therapy to improve upper extremity function after stroke. J Rehabil Med. 2011 Jun;43(7):619–25.

451.
Wee SK, Hughes A-M, Warner MB, Brown S, Cranny A, Mazomenos EB, et al. Effect of Trunk Support on Upper Extremity Function in People With Chronic Stroke and People Who Are Healthy. Phys Ther. 2015 Aug;95(8):1163–71.

452.
Weiss T, Sens E, Teschner U, Meissner W, Preul C, Witte OW, et al. Deafferentation of the affected arm: a method to improve rehabilitation? Stroke. 2011 May;42(5):1363–70.

453.
Winstein CJ, Wolf SL, Dromerick AW, Lane CJ, Nelsen MA, Lewthwaite R, et al. Interdisciplinary Comprehensive Arm Rehabilitation Evaluation (ICARE): a randomized controlled trial protocol. BMC Neurol. 2013;13:5.

454.
Winter JM, Crome P, Sim J, Hunter SM. Effects of mobilization and tactile stimulation on chronic upper-limb sensorimotor dysfunction after stroke. Arch Phys Med Rehabil. 2013 Apr;94(4):693–702.

455.
Woldag H, Renner C, Hummelsheim H. Isotonic and isometric contractions exert the same amount of corticomotor system excitability in healthy subjects and patients after stroke. J Rehabil Med. 2008 Feb;40(2):107–11.

456.
Woldag H, Stupka K, Hummelsheim H. Repetitive training of complex hand and arm movements with shaping is beneficial for motor improvement in patients after stroke. J Rehabil Med. 2010 Jun;42(6):582–7.

457.
Wolf SL, Milton SB, Reiss A, Easley KA, Shenvi NV, Clark PC. Further assessment to determine the additive effect of botulinum toxin type A on an upper extremity exercise program to enhance function among individuals with chronic stroke but extensor capability. Arch Phys Med Rehabil. 2012 Apr;93(4):578–87.

458.
Wolf SL, Thompson PA, Winstein CJ, Miller JP, Blanton SR, Nichols-Larsen DS, et al. The EXCITE stroke trial: comparing early and delayed constraint-induced movement therapy. Stroke. 2010 Oct;41(10):2309–15.

459.
Wolf SL, Winstein CJ, Miller JP, Taub E, Uswatte G, Morris D, et al. Effect of constraint-induced movement therapy on upper extremity function 3 to 9 months after stroke: the EXCITE randomized clinical trial. JAMA. 2006 Nov 1;296(17):2095–104.

460.
Woodbury ML, Howland DR, McGuirk TE, Davis SB, Senesac CR, Kautz S, et al. Effects of trunk restraint combined with intensive task practice on poststroke upper extremity reach and function: a pilot study. Neurorehabil Neural Repair. 2009 Jan;23(1):78–91.

461.
Wu CW, Seo H-J, Cohen LG. Influence of electric somatosensory stimulation on paretic-hand function in chronic stroke. Arch Phys Med Rehabil. 2006 Mar;87(3):351–7.

462.
Wu C, Chen C, Tang SF, Lin K, Huang Y. Kinematic and clinical analyses of upper-extremity movements after constraint-induced movement therapy in patients with stroke: a randomized controlled trial. Arch Phys Med Rehabil. 2007 Aug;88(8):964–70.

463.
Wu C, Chen Y, Chen H, Lin K, Yeh I -lin. Pilot trial of distributed constraint-induced therapy with trunk restraint to improve poststroke reach to grasp and trunk kinematics. Neurorehabil Neural Repair. 2012 Apr;26(3):247–55.

464.
Wu C, Chen Y, Lin K, Chao C, Chen Y. Constraint-induced therapy with trunk restraint for improving functional outcomes and trunk-arm control after stroke: a randomized controlled trial. Phys Ther. 2012 Apr;92(4):483–92.

465.
Wu C, Chou S, Kuo M, Chen C, Lu T, Fu Y. Effects of object size on intralimb and interlimb coordination during a bimanual prehension task in patients with left cerebral vascular accidents. Motor Control. 2008 Oct;12(4):296–310.

466.
Wu C-Y, Hsieh Y-W, Lin K-C, Chuang L-L, Chang Y-F, Liu H-L, et al. Brain reorganization after bilateral arm training and distributed constraint-induced therapy in stroke patients: a preliminary functional magnetic resonance imaging study. Chang Gung Med J. 2010 Dec;33(6):628–38.

467.
Yamada N, Kakuda W, Kondo T, Mitani S, Shimizu M, Abo M. Local muscle injection of botulinum toxin type a synergistically improves the beneficial effects of repetitive transcranial magnetic stimulation and intensive occupational therapy in post-stroke patients with spastic upper limb hemiparesis. Eur Neurol. 2014;72(5-6):290–8.

468.
Yamaguchi T, Tanabe S, Muraoka Y, Imai S, Masakado Y, Hase K, et al. Effects of integrated volitional control electrical stimulation (IVES) on upper extremity function in chronic stroke. Keio J Med. 2011;60(3):90–5.

469.

Yamamoto T, Katayama Y, Watanabe M, Sumi K, Obuchi T, Kobayashi K, et al. Changes in motor function induced by chronic motor cortex stimulation in post-stroke pain patients. Stereotact Funct Neurosurg. 2011;89(6):381–9.

470. Yang C-L, Lin K-C, Chen H-C, Wu C-Y, Chen C-L. Pilot comparative study of unilateral and bilateral robot-assisted training on upper-extremity performance in patients with stroke. Am J Occup Ther. 2012 Apr;66(2):198–206.

471. Yavuzer G, Selles R, Sezer N, Sütbeyaz S, Bussmann JB, Köseoğlu F, et al. Mirror therapy improves hand function in subacute stroke: a randomized controlled trial. Arch Phys Med Rehabil. 2008 Mar;89(3):393–8.

472. Yoo I, Jung M, Yoo E, Park S, Park J, Lee J, et al. Effect of specialized task training of each hemisphere on interlimb transfer in individuals with hemiparesis. NeuroRehabilitation. 2013;32(3):609–15.

473. Yozbatiran N, Donmez B, Kayak N, Bozan O. Electrical stimulation of wrist and fingers for sensory and functional recovery in acute hemiplegia. Clin Rehabil. 2006 Jan;20(1):4–11.

474. Ziherl J, Novak D, Olenšek A, Mihelj M, Munih M. Evaluation of upper extremity robot-assistances in subacute and chronic stroke subjects. J Neuroeng Rehabil. 2010;7:52.

475. Zimerman M, Heise KF, Hoppe J, Cohen LG, Gerloff C, Hummel FC. Modulation of training by single-session transcranial direct current stimulation to the intact motor cortex enhances motor skill acquisition of the paretic hand. Stroke. 2012 Aug;43(8):2185–91.

476. Zittel S, Weiller C, Liepert J. Reboxetine improves motor function in chronic stroke. A pilot study. J Neurol. 2007 Feb;254(2):197–201.

477. Zittel S, Weiller C, Liepert J. Citalopram improves dexterity in chronic stroke patients. Neurorehabil Neural Repair. 2008 Jun;22(3):311–4.
